# Clinical phenotypes and quality of life to define post-COVID-19 syndrome: a cluster analysis of the multinational, prospective ORCHESTRA cohort

**DOI:** 10.1016/j.eclinm.2023.102107

**Published:** 2023-07-21

**Authors:** Elisa Gentilotti, Anna Górska, Adriana Tami, Roy Gusinow, Massimo Mirandola, Jesús Rodríguez Baño, Zaira R. Palacios Baena, Elisa Rossi, Jan Hasenauer, Iris Lopes-Rafegas, Elda Righi, Natascia Caroccia, Salvatore Cataudella, Zeno Pasquini, Thomas Osmo, Lidia Del Piccolo, Alessia Savoldi, Samir Kumar-Singh, Fulvia Mazzaferri, Maria Giulia Caponcello, Gerolf de Boer, Gabriel Levy Hara, ORCHESTRA Study Group, Mariana Nunes Pinho Guedes, Gaia Maccarrone, Maria Diletta Pezzani, Marcella Sibani, Ruth Joanna Davies, Stefania Vitali, Giorgia Franchina, Giorgia Tomassini, Concetta Sciammarella, Riccardo Cecchetto, Davide Gibellini, Chiara Konishi De Toffoli, Giulia Rosini, Chiara Perlini, Marco Meroi, Filippo Cioli Puviani, Daniele Fasan, Claudio Micheletto, Stefania Montemezzi, Nicolò Cardobi, Gianluca Vantini, Gloria Mazzali, Giovanni Stabile, Maddalena Marcanti, Marco Pattaro Zonta, Deborah Calì, Anna Mason, Cinzia Perlini, Paolo Gisondi, Maria Mongardi, Simona Sorbello, Karin I. Wold, María F. Vincenti-González, Alida C.M. Veloo, Valerie P.R. Harmsma, Daniele Pantano, Margriet van der Meer, Lilli Gard, Erley F. Lizarazo, Marjolein Knoester, Alex W. Friedrich, Hubert G.M. Niesters, Pierluigi Viale, Domenico Marzolla, Federica Cosentino, Michela Di Chiara, Giacomo Fornaro, Cecilia Bonazzetti, Beatrice Tazza, Alice Toschi, Oana Vetamanu, Maria Eugenia Giacomini, Fabio Trapani, Lorenzo Marconi, Luciano Attard, Sara Tedeschi, Liliana Gabrielli, Tiziana Lazzarotto, Paula Olivares, Javier Castilla, Javier Vélez, Virginia Almadana, Lucía Martín-Barrera, Ana Belén Martín-Gutiérrez, David Gutiérrez-Campos, Marta Fernández-Regaña, Ana Silva-Campos, Patricia Fernández-Riejos, M. Isabel García-Sánchez, Carla V. Giuliano, Carlota López, Gabriela Neumann, Julieta Camporro, Lautaro de Vedia, Hugo Agugliaro, Gabriella Scipione, Chiara Dellacasa, Balasubramanian Chandramouli, Silvia Gioiosa, Juan Mata Naranjo, Maurizio Ortali, Angelina Konnova, Akshita Gupta, Mathias Smet, An Hotterbeekx, Matilda Berkell, Elisa Sicuri, Delphine Bachelet, Lila Bouadma, Minerva Cervantes-Gonzalez, Anissa Chair, Charlotte Charpentier, Léo Chenard, Diane Descamps, Hang Doan, Xavier Duval, Marina Esposito-Farese, Isabelle Hoffmann, Ouifiya Kafif, Quentin Le Hingrat, Sophie Letrou, France Mentré, Marion Schneider, Coralie Tardivon, Jean-Francois Timsit, Sarah Tubiana, Amal Abrous, Sandrine Couffin-Cadiergues, Fernanda Dias Da Silva, Hélène Esperou, Ikram Houas, Salma Jaafoura, Aurélie Papadopoulos, Severine Ansart, Adrien Auvet, Firouzé Bani-Sadr, L. Bernard, François Bissuel, Elisabeth Botelho-Nevers, Damien Bouhour, André Cabié, Pauline Caraux Paz, Christian Chidiac, Catherine Chirouze, Tomasz Chroboczek, Hugues Cordel, Roxane Courtois, Nathalie De Castro, Sylvain Diamamntis, Jean-Luc Diehl, Felix Djossou, Céline Dorival, Olivier Epaulard, Valerie Gaborieau, François Goehringer, Marie Gousseff, Simon Jamard, Cedric Joseph, Karine Lacombe, Soizic Le Mestre, Vincent Le Moing, Jean-Daniel Lelievre, Olivier Lesens, M. Machado, Mylène Maillet, Victoria Manda, Guillaume Martin-Blondel, Martin Martinot, Vanina Meysonnier, Jean-Michel Molina, Eric Oziol, Vincent Pestre, Lionel Piroth, Julien Poissy, Christian Rabaud, François Raffi, Blandine Rammaert, Christophe Rapp, Stanislas Rebaudet, Pierre-Marie Roger, Damien Roux, Eric Senneville, Pierre Tattevin, Aurélie Wiedemann, David Zucman, Pasquale De Nardo, Surbhi Malhotra, Lorenzo Maria Canziani, Jade Ghosn, Aline-Marie Florence, Nadhem Lafhej, Bernardina T.F. van der Gun, Maddalena Giannella, Cédric Laouénan, Evelina Tacconelli

**Affiliations:** a Infectious Disease, Department of Diagnostics and Public Health, University of Verona, Verona, Italy; b University of Groningen, University Medical Center Groningen, Department of Medical Microbiology and Infection Prevention, Groningen, The Netherlands; c The Life & Medical Sciences Institute (LIMES), University of Bonn-Institute for Computational Biology, Helmholtz Munich; Research Center for Environmental Health, Neuherberg, Germany; d Unidad Clínica de Enfermedades Infecciosas y Microbiología, Hospital Universitario Virgen Macarena, Departamento de Medicina, Universidad de Sevilla, Spain; e CINECA Interuniversity Consortium, Bologna, Italy; f Barcelona Institute for Global Health (ISGlobal), Hospital Clínic, University of Barcelona, Spain; g Department of Medical and Surgical Sciences, Alma Mater Studiorum, University of Bologna, Bologna, Italy; h Infectious Diseases Unit, IRCCS Azienda Ospedaliero-Universitaria di Bologna, Bologna, Italy; i Centre Informatique National de l'Enseignement Supérieur CINES, France; j Department of Neurosciences, Biomedicine and Movement Sciences, University of Verona, Verona, Italy; k Molecular Pathology Group, Cell Biology & Histology, and Laboratory of Medical Microbiology, Vaccine & Infectious Disease Institute, Faculty of Medicine, University of Antwerp, Antwerp, Belgium; l Instituto Alberto C. Taquini de Investigaciones en Medicina Traslacional, Facultad de Medicina, Universidad de Buenos Aires, Argentina; m Université Paris Cité, INSERM IAME UMR 1137, Paris, France; n AP-HP Nord, Hôpital Bichat, Department of Infectious and Tropical Diseases, Paris, France; o AP-HP Nord, Hôpital Bichat, Department of Epidemiology Biostatistics and Clinical Research, Paris, France; p Instituto de Biomedicina de Sevilla (IBiS)/CSIC, Seville, Spain; q CIBERINFEC, Instituto de Salud Carlos III, Madrid, Spain

**Keywords:** COVID-19, SARS-CoV-2, Long-term sequelae, Prediction model, Post-COVID syndrome

## Abstract

**Background:**

Lack of specific definitions of clinical characteristics, disease severity, and risk and preventive factors of post-COVID-19 syndrome (PCS) severely impacts research and discovery of new preventive and therapeutics drugs.

**Methods:**

This prospective multicenter cohort study was conducted from February 2020 to June 2022 in 5 countries, enrolling SARS-CoV-2 out- and in-patients followed at 3-, 6-, and 12-month from diagnosis, with assessment of clinical and biochemical features, antibody (Ab) response, Variant of Concern (VoC), and physical and mental quality of life (QoL). Outcome of interest was identification of risk and protective factors of PCS by clinical phenotype, setting, severity of disease, treatment, and vaccination status. We used SF-36 questionnaire to assess evolution in QoL index during follow-up and unsupervised machine learning algorithms (principal component analysis, PCA) to explore symptom clusters. Severity of PCS was defined by clinical phenotype and QoL. We also used generalized linear models to analyse the impact of PCS on QoL and associated risk and preventive factors. CT registration number: NCT05097677.

**Findings:**

Among 1796 patients enrolled, 1030 (57%) suffered from at least one symptom at 12-month. PCA identified 4 clinical phenotypes: chronic fatigue-like syndrome (CFs: fatigue, headache and memory loss, 757 patients, 42%), respiratory syndrome (REs: cough and dyspnoea, 502, 23%); chronic pain syndrome (CPs: arthralgia and myalgia, 399, 22%); and neurosensorial syndrome (NSs: alteration in taste and smell, 197, 11%). Determinants of clinical phenotypes were different (all comparisons p < 0.05): being female increased risk of CPs, NSs, and CFs; chronic pulmonary diseases of REs; neurological symptoms at SARS-CoV-2 diagnosis of REs, NSs, and CFs; oxygen therapy of CFs and REs; and gastrointestinal symptoms at SARS-CoV-2 diagnosis of CFs. Early treatment of SARS-CoV-2 infection with monoclonal Ab (all clinical phenotypes), corticosteroids therapy for mild/severe cases (NSs), and SARS-CoV-2 vaccination (CPs) were less likely to be associated to PCS (all comparisons p < 0.05). Highest reduction in QoL was detected in REs and CPs (43.57 and 43.86 vs 57.32 in PCS-negative controls, p < 0.001). Female sex (p < 0.001), gastrointestinal symptoms (p = 0.034) and renal complications (p = 0.002) during the acute infection were likely to increase risk of severe PCS (QoL <50). Vaccination and early treatment with monoclonal Ab reduced the risk of severe PCS (p = 0.01 and p = 0.03, respectively).

**Interpretation:**

Our study provides new evidence suggesting that PCS can be classified by clinical phenotypes with different impact on QoL, underlying possible different pathogenic mechanisms. We identified factors associated to each clinical phenotype and to severe PCS. These results might help in designing pathogenesis studies and in selecting high-risk patients for inclusion in therapeutic and management clinical trials.

**Funding:**

The study received funding from the Horizon 2020 ORCHESTRA project, grant 101016167; from the 10.13039/501100001826Netherlands Organisation for Health Research and Development (ZonMw), grant 10430012010023; from 10.13039/501100001677Inserm, REACTing (REsearch & ACtion emergING infectious diseases) consortium and the French Ministry of Health, grant PHRC 20-0424.

**Research in context**

**Evidence before this study**

We performed a review of the literature to identify existing studies on clinical characterization of PCS and impact on quality of life (QoL) (Supplementary Section 4). Due to the generic definition and lack of pathognomonic characterization, prevalence of disease greatly varies among studies. Fatigue was the most frequent symptom, ranging from 10% to 53%. Respiratory disorders were also frequently reported (8%–37%), followed by cognitive impairment (including brain fog, difficulty in thinking, poor attention, memory loss, and confusion; 6%–35%) (Supplementary Table S8). The majority of studies showed a lower QoL in patients experiencing PCS compared with the general population, although a high heterogeneity in terms of time of assessment and type of tests used was observed (Supplementary Table S9). Few studies found that COVID-19 symptoms could be classified in clusters of symptoms. Risk and preventive factors by cluster have not been yet identified.

**Added value of this study**

The prospective design of the ORCHESTRA cohort allowed for an extraordinary granularity of data. PCS determinants could be explored from different perspectives and through multiple variables, including medical history, concomitant treatments, acute infection characteristics, VoC, and variation in time of serology and biochemistry patterns. The multiple follow-ups reduced the risk of missing essential information such as breakthrough infections and vaccination status. Using machine learning and principal component analysis, we identified four clinical phenotypes of the PCS and associated factors, and proposed the first definition for clinical severity of the PCS, based on its impact on quality of life. The result of the ORCHESTRA study is a comprehensive, multifaceted analysis adding new evidence on PCS from definition to impact on patients’ quality of life.

**Implications of all the available evidence**

The evidence recognised in the ORCHESTRA project, in terms of PCS determinants and severity of disease, can support the early identification of patients at higher risk of development of PCS and therefore drive implementation of appropriate follow-up management protocols. The confirmation that vaccination has a substantial role in preventing chronic fatigue syndrome post SARS-CoV-2 acute infection could further support public awareness campaign and policy. Early identification of patients at risk could also have a pivotal role in improving patients’ selection in clinical trials for new preventive treatment of PCS and epidemiological studies assessing the burden of PCS.

## Introduction

The prevalence of post-COVID-19 syndrome (PCS, also referred as “post-acute sequelae of COVID-19”, “long COVID-19”, or “persistent COVID-19”) varies widely between 8% and 70% depending on definition, population, symptom assessment method, number of symptoms, and time points assessed.^1^^,^^2^ According to the WHO definition, PCS occurs in individuals with a history of probable or confirmed SARS-CoV-2 infection, usually within 3 months from diagnosis, with symptoms that last for at least two months and cannot be explained by an alternative diagnosis.^3^ Around 65 million individuals were estimated to suffer from PCS, based on a conservative estimated incidence of 10% of infected people and more than 651 million documented COVID-19 cases.^4^ Common symptoms include fatigue, shortness of breath, and cognitive dysfunction.^5^^,^^6^ Symptoms may be new onset following initial recovery from an acute COVID-19 episode or persist from the initial illness, and may also fluctuate or relapse over time.^3^ Studies showed that patients reporting persistence of symptoms after acute SARS-CoV-2 infection also experience reduced QoL.^7^^,^^8^

More recently, increasing clinical experience and studies, suggest the possibility that the symptoms of PCS might have a cluster distribution.^9^, ^10^, ^11^ Several hypotheses have been suggested to explain the possible mechanisms leading to the persistence of symptoms, including uncontrolled immune responses, inflammatory damage, coagulation alteration, viral direct effects, and virus interactions with host microbiome and virome.^12^^,^^13^

ORCHESTRA (connecting European cohorts to increase common and effective response to SARS-CoV-2 pandemic) is a H2020 project, including 37 Partners from 15 countries, aiming at tackling the Coronavirus pandemic to establish an international large-scale-cohort to generate rigorous evidence in the field of prevention and treatment of SARS-CoV-2 infection.^14^, ^15^, ^16^ The ORCHESTRA work package 2 (WP2) implemented a multi-country prospective observational cohort of patients to define PCS by periodically assessing clinical, virological, biochemical, and immunological aspects and QoL from diagnosis of SARS-CoV-2 infection up to one year follow-up.

The objectives of the study were: to assess prevalence of PCS according to the WHO definition^3^ and cluster of symptoms; to investigate factors associated to PCS by clinical phenotype, comorbidities, severity and treatment of acute infection (including early treatments), vaccination status, VoC, and anti-S Ab titer; and to analyse severity of PCS by clinical phenotypes and QoL.

## Methods

### Study design and participants

The ORCHESTRA WP2 includes six prospective cohorts from 56 centers in five countries (France, Italy, the Netherlands, Spain, and Argentina) of patients with SARS-CoV-2 infection followed from February 2020 to June 2022. Epidemiological and clinical characteristics of cohorts are summarized in the Supplementary Tables S1–S3. The protocol is available at the ORCHESTRA website.^14^ In- and out-patients aged >14 years old with a laboratory-confirmed SARS-CoV-2 infection were included in the study after written informed consent and followed at 3-, 6-, and 12-month post-infection at an outpatient clinic or at the patients’ home. Each follow-up visit combined a clinical assessment, performed by qualified medical staff, and laboratory tests, including biochemical parameters and serology (Supplementary Section 2, Supplementary Table S4). Nasopharyngeal swabs were performed to define the VoC at baseline and repeated only in case of positive sampling after 30 days since infection’s diagnosis. VoC and serological analysis were performed at central laboratory of Antwerp or at local laboratories (the Netherlands, France, and Argentina) using homogenised protocols (Supplementary Section 2, Supplementary Table S4). Data assessed at 3- and 6-month follow-up are not reported here, with a few exceptions, related to specific research questions.

Study data were collected and managed using REDCap electronic data capture tool (Research Electronic Data CAPpture). Since the cohorts in France and in the Netherlands started before the ORCHESTRA project was financed (in February and March 2020, respectively), data from these two cohorts went through a post-data collection harmonization process under the supervision of the Charité, Universitätsmedizin Berlin and transformation by the Centre Informatique National de l’Enseignement Superieur.^15^^,^^16^ Data collected at baseline included date of symptom onset and diagnosis, duration of symptoms, demographic characteristics, comorbidities, clinical presentation, treatment of acute infection, hospitalization, admission to ICU, and post-acute infection complications. From February 2021 to June 2022, recommendations for early treatment (e.g., anti-SARS-CoV-2 out-patient therapy within the first five days of onset of symptoms, according to national recommendations) included three monoclonal antibodies (bamlanivimab, bamlanivimab/etesevimab, and casirivimab/imdevimab). Occurrence of new medical events, vital signs and physical examination, laboratory parameters, and vaccination status were collected at each time point. A symptom was considered to be associated to SARS-CoV-2 infection, if newly diagnosed after acute infection or if a significant worsening in terms of severity and/or presentation of the symptom was registered after acute infection in case of pre-existing medical conditions. QoL was assessed through the physical component score and the mental component score of the SF-36 questionnaire^17^^,^^18^ at 6- and 12-month after acute infection. The questionnaires were scored using the PRO CoRE software developed by QualityMetrics, which applies US1998 norms. Definition of suboptimal score was based on the 25th percentile of the distribution for patients not reporting symptoms at the 12-month assessment. A poor QoL was defined for a score below 50. Severity of PCS was analysed assessing the impact of each cluster of symptoms and by their combination on QoL. The study was approved by all local ethics committees (Supplementary Table S2) and by the coordinator center (3199CESC).

### Statistical analysis

Means and standard deviations (SD) were calculated for continuous variables and frequency tables for categorical variables. For the univariable analysis, crude odds ratios (OR) with 95% confidence intervals (95% CI) of the categorical variables were shown with corresponding p-values. Bonferroni correction was applied to account for multiple comparisons in univariable analysis. To test differences across categories, the Chi-square test was applied for categorical data. Otherwise, the non-parametric Kruskal–Wallis test was used for continuous data that were not normally distributed. Parametric tests have been applied, being the sample size large enough. To determine factors associated to the primary endpoints we assessed their correlation with the continuous and categorical covariates and interaction terms using methods from single- and multi-variable risk factor analysis. Specifically, we applied logistic regression models by considering a generalized linear model (GLM) with log-odds linking function (i.e., Bernoulli distribution). Controls were patients with no new symptom persisting more than 90 days after SARS-CoV-2 infection. Variables resulting to be significant at univariable analysis (as defined based on corrected p-value = 0.001/number of tests) were selected to be included in the logistic regression models. Model selection was done by evaluating the AIC (Akaike Information Criterion) of the models that use all possible combinations (subsets) of factors deemed significant, adjusting for age and sex as additional risk factors. Through AIC methodology, all possible significant risk factors were taken into account, avoiding overfitting with a large number of covariates. A regression was run to account for statistical difference (p < 0.05) between the outcome and the variables of interest. This methodology was applied to assess the pattern of missing data and exclude that it is correlated with any systematic variable. Unsupervised machine learning algorithm, principal component analysis (PCA), was used to study clusters of symptoms and logistic regression to perform an analysis of factors associated with each of the clusters. Details of the PCA are provided in the Supplementary Methods, Supplementary Figure S1). We applied multivariable logistic regression to determine risk and preventive factors associated with severe PCS. Performance of the model was measured by running 8-folds cross validation using the lowest AIC logistic regression model found. The accuracy displays the number of correctly identified patient outcome over the entire cohort used. As some phenotypes occur rarely and therefore are underrepresented when testing the model, a balanced accuracy is used, measuring a weighted average. This is calculated by halving the combined sensitivity and specificity readings (sensitivity + specificity)/2). Mean accuracy and mean balanced accuracy of the 8-fold is reported.

All analyses were performed according to the WHO definition of PCS (i.e., presence of at least one unexplained symptom) and by the clinical phenotypes identified in our cohort. The statistical analysis was performed using R version 4.1.3 and using the R package stats (version 3.6.2).

### Role of the funding source

The funder of the study had no role in study design, data collection, data analysis, data interpretation, or writing of the report.

## Results

### Study design and participants

Overall, 1796 patients completed the 12-month follow-up and were included in the analysis. Most patients were male (1016, 57%) and aged between 41 and 60 years (774, 43%, average ± SD: 57.2 ± 14.9). Cardiovascular diseases (710, 40%) were the most frequently reported underlying clinical conditions. Two hundred and eighty-three patients (16%) had a breakthrough infection, 1081 (60%) received at least one dosage of SARS-CoV-2 vaccination after the infection, and 123 (7%) received monoclonal antibodies administered as early treatment for SARS-CoV-2 infection. Patients enrolled in hospitals were 1267 (71%) and 419 (33%) of them required ICU admission.

### Prevalence and factors associated with PCS defined as presence of at least one symptom at month-12

**Fig. 1:**
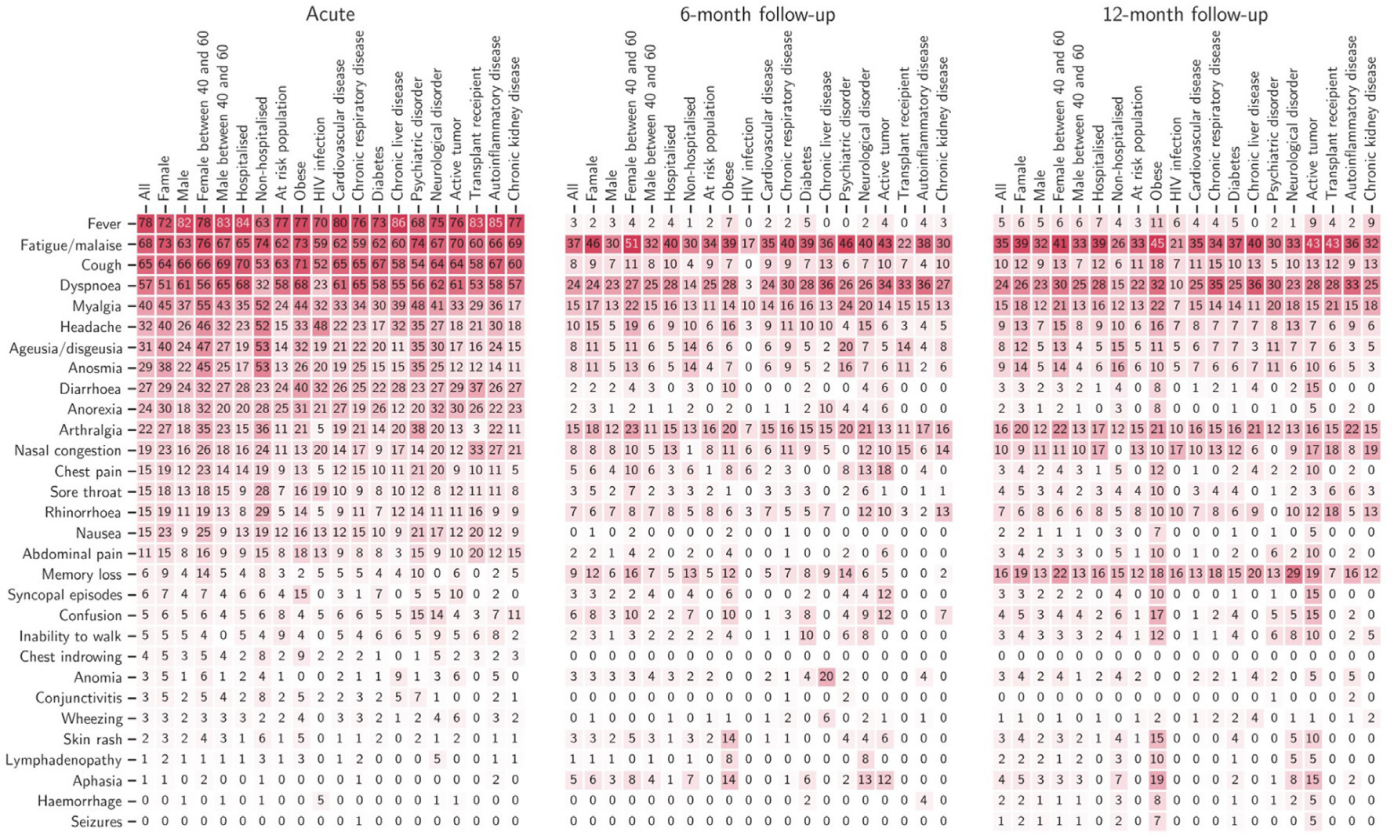
**Relative frequency of symptoms across the population subgroups according to the demographic features and comorbidities.** Each value corresponds to the percentage of the patient-group defined by x-axis, who presents with a symptom outlined on y-axis, at described time point. E.g., fever was reported by 78% of the overall patients in the acute phase, but only by 3% in the 6-month follow-up. Variables in the x-axis were selected based on clinical meaningfulness. Obese: BMI ≥30; at risk population: age >65 years old and/or at least one of the following: BMI ≥30, chronic kidney disease, diabetes, HIV infection, cardiovascular disease, chronic respiratory diseases, chronic liver disease, neurological disorder.

At 12-month assessment, 1030 (57%) out of 1796 patients reported suffering from at least one COVID-related symptom. Fig. 1 displays a selection of symptoms’ distribution by population subgroups and comorbidities. Details of the PCA are provided in Supplementary Figure S1. Univariable analyses of factors associated to PCS are reported in the Supplementary Table S5 and Supplementary Figure S2. The SF-36 questionnaire was completed by 1193 patients at 12-month follow-up and of these, 527 patients had a score below 50. Patients with at least one symptom had a lower score in physical (47.67 vs 57.32, p < 0.001) and mental components (46.77 vs 54.22, p < 0.001) when compared with patients with history of SARS-CoV-2 with no symptoms (Supplementary Section 3, Supplementary Figures S3 and S4). Blood test parameters analysis revealed that patients with at least one complaint at 12-month assessment had higher CRP, PCT and AST levels during the acute infection compared with patients without PCS (Supplementary Figure S2).

Applying multivariable analysis, neurological symptoms during the acute infection (OR: 2.16, 95% CI: 1.56–3.01, p < 0.001) and being female (OR: 1.81, 95% CI: 1.34–2.46, p < 0.001) were independently associated with PCS at month-12. Patients receiving early treatment for COVID-19 with monoclonal antibodies were less likely to develop PCS (OR: 0.19, 95% CI: 0.11–0.33, p < 0.001) (Table 1). Variables independently associated with a suboptimal score for QoL (<50) at month-12 were: female sex (OR: 3.08, 95% CI: 2.22–4.32, p < 0.001), advanced age (OR: 1.02, 95% CI: 1.01–1.04, p = 0.001), hospital admission (OR: 2.36, 95% CI: 1.49–3.75, p < 0.001), pre-existing chronic respiratory diseases (OR: 2.39, 95% CI: 1.59–3.61, p < 0.001), diabetes (OR: 1.83, 95% CI: 1.03–3.32, p = 0.04), respiratory symptoms (OR: 1.82, 95% CI: 1.04–3.28, p = 0.04), and renal complications (OR: 2.33, 95% CI: 1.11–5.12, p = 0.03) during the acute infection (Table 2).

**Table 1:** Multivariable analysis of factors associated with at least one symptom at 12-month assessment (accuracy 0.66; balanced accuracy 0.65).

Variable	At least 1 symptom according to WHO definition of post-COVID-19 syndrome (N = 822)			
	OR	95% CI		p-value
		LB	UB	
Female sex	1.81	1.34	2.46	**<0.001**
Age	1.01	1.00	1.02	0.20
Neurological symptoms^a^	2.16	1.56	3.01	**<0.001**
Hospital admission^a^	0.62	0.37	1.02	0.06
Early therapy (monoclonal antibody)^b^	0.19	0.11	0.33	**<0.001**
Oxygen therapy^a^	1.54	0.96	2.46	0.07

OR: odd ratio; CI: confident interval; LB: lower bound; UB: upper bound; WHO: World Health Organization.

The significant associations denoted with a bold font refer to p-values <0.05.

a During the acute infection.

b Monoclonal antibodies administered within the first three days of symptoms in high risk patients (see description in the Methods section of the manuscript): bamlanivimab, bamlanivimab/etesevimab, casirivimab/imdevimab.

**Table 2:** Multivariable analysis of factors associated with suboptimal score at SF-36 questionnaire physical score (<50) at 12-month assessment (accuracy 0.64; balanced accuracy 0.64).

Variable	SF-36 Physical component score <50 (N = 810)			
	OR	95% CI		p-value
		LB	UB	
Female sex	3.08	2.22	4.32	**<0.001**
Age	1.02	1.01	1.04	**0.001**
First wave^a^	1.38	0.96	2.00	0.08
Hospital admission^b^	2.36	1.49	3.75	**<0.001**
Early therapy (monoclonal antibody)^c^	0.65	0.28	1.40	0.28
Chronic respiratory disease^d^	2.39	1.59	3.61	**<0.001**
Diabetes	1.83	1.03	3.32	**0.04**
Cardiovascular disease^e^	1.34	0.95	1.90	0.10
Transplant	2.17	0.69	7.76	0.20
Respiratory symptoms^b^	1.82	1.04	3.28	**0.04**
Renal events^b^	2.33	1.11	5.12	**0.04**
Anti-viral therapy^f^	0.82	0.55	1.23	0.34

OR: odd ratio; CI: confident interval; LB: lower bound; UB: upper bound; SF-36: short form health survey 36.

The significant associations denoted with a bold font refer to p-values <0.05.

a First wave: SARS-CoV-2 infection before September 2020.

b During the acute infection.

c Monoclonal antibodies administered within the first three days of symptoms in high risk patients (see description in the Methods section of the manuscript): bamlanivimab, bamlanivimab/etesevimab, casirivimab/imdevimab.

d Including asthma, chronic obstructive pulmonary disease, obstructive sleep apnoea syndrome, pulmonary hypertension, restrictive lung diseases.

e Including congestive heart failure, coronary heart diseases, hypertension.

f It does not include (being not available at the time of SARS-CoV-2 diagnosis of this cohort) nirmatrelvir/ritonavir and molnupinavir; it includes ribavirin, darunavir, lopinavir/ritonavir, interferon alfa, interferon beta, neuraminidase inhibitors, favipiravir, remdesivir, camostat, atazanavir.

### Prevalence and factors for PCS defined by clinical phenotypes

**Fig. 2:**
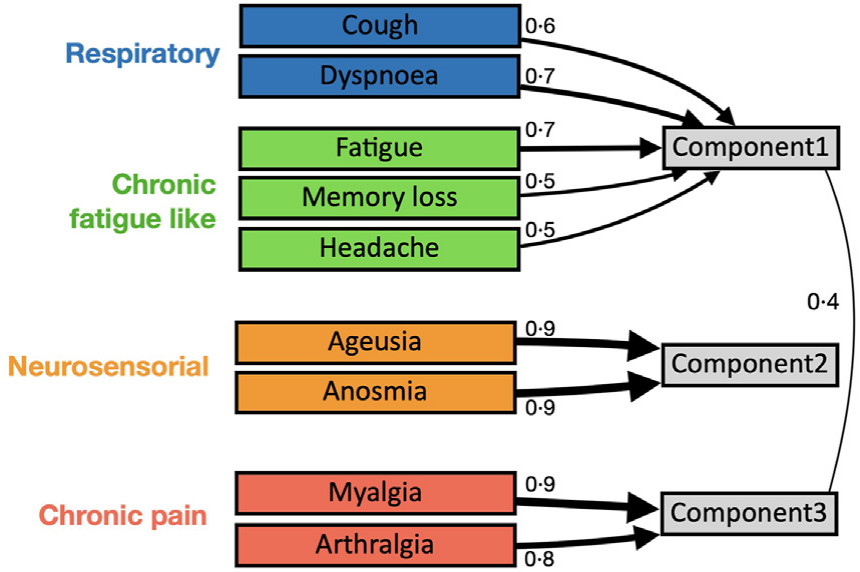
**Clusters of symptoms according to principal component analysis (PCA).** The numbers near the arrows are the loadings (only loadings >0.4 are depicted). Component 1, 2, and 3 denote the obliquely transformed components.

Fig. 2 and Supplementary Figure S1 summarize application of the PCA to analyse clusters of symptoms. Among 1030 patients with at least one symptom at 12-month, we identified four clinical phenotypes: chronic fatigue-like syndrome (CFs: fatigue, headache, and memory loss; 757, 42%), respiratory syndrome (REs: cough and dyspnoea; 502, 23%); chronic pain syndrome (CPs: arthralgia and myalgia; 399, 22%); and neurosensorial syndrome (NSs: alteration in taste and smell; 197, 11%). One hundred and fifty-five patients (8%) had more than one cluster of symptoms. Univariable analysis is reported in the Supplementary Figure S5.

**Fig. 3:**
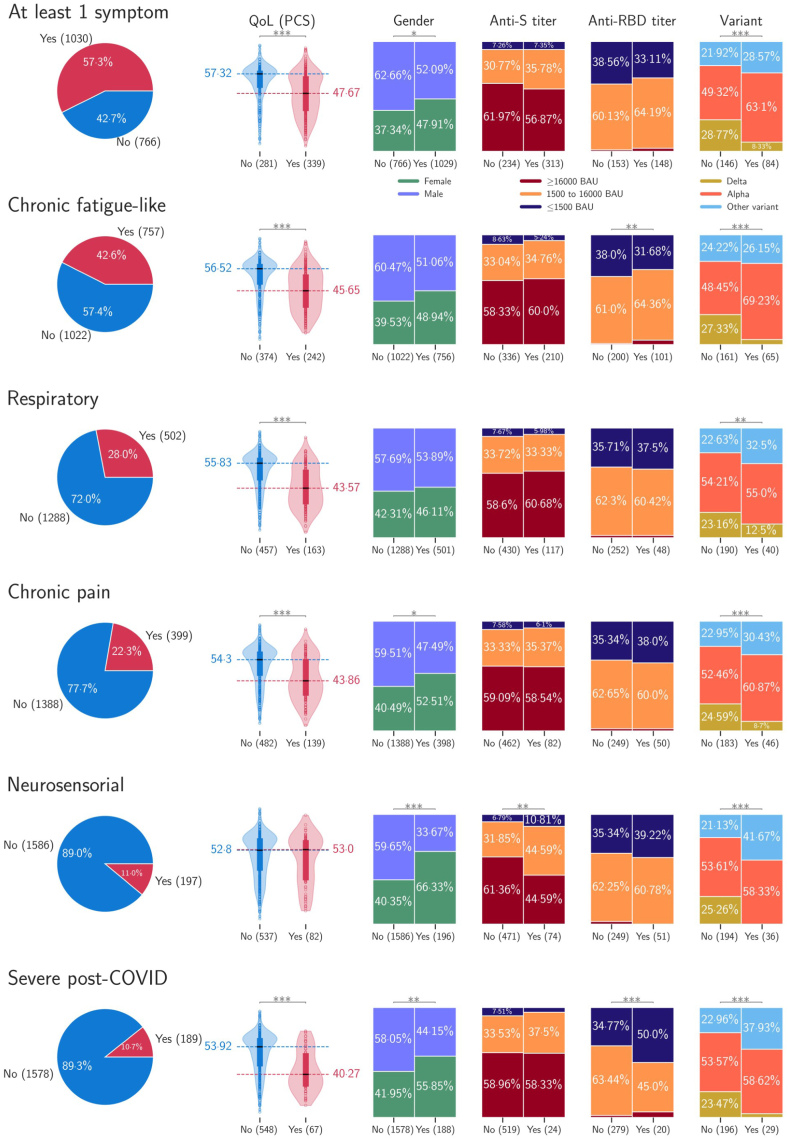
**Comparison among distribution of gender, immune response (anti-S and anti-RBD titers) and SARs-CoV-2 variants by WHO definition, clinical phenotypes, and severity of post-COVID-19 syndrome.** The numbers in brackets denote the number of patients.

Using multivariable analysis, being female was associated with a higher risk of NSs (OR: 2.25, 95% CI: 1.35–3.85, p = 0.002), CPs (OR: 1.94, 95% CI: 1.25–3.05, p = 0.004), and CFs (OR: 2.14, 95% CI: 1.53–3.02, p < 0.001); presence of neurological symptoms at SARS-CoV-2 diagnosis increased the risk of NSs (OR: 40.76; 95% CI: 8.85–724.2, p < 0.001), REs (OR: 1.71, 95% CI: 1.14–2.63, p = 0.01), and CFs (OR: 1.61, 95% CI: 1.11–2.35, p = 0.01); gastroenterological symptoms (OR: 1.48, 95% CI: 1.06–2.07, p = 0.02) increased the risk of CFs; chronic respiratory diseases (OR: 1.66, 95% CI: 1.05–2.59, p = 0.03) were associated with REs (Table 3). Fig. 3 summarizes distribution of risks according to the WHO definition^3^ or the ORCHESTRA clinical phenotypes of PCS by gender, serology, VoC, and QoL.

**Table 3:** Multivariable analysis of factors by clinical phenotypes of the post-COVID-19 syndrome at 12-month of SARS-CoV-2 diagnosis.

Variable	Respiratory syndrome N = 1789				Neurosensorial syndrome N = 1782				Chronic pain syndrome N = 1726				Chronic fatigue-like syndrome N = 1779			
	OR	95% CI		p-value	OR	95% CI		p-value	OR	95% CI		p-value	OR	95% CI		p-value
		LB	UB			LB	UB			LB	UB			LB	UB	
Female sex	1.19	0.83	1.70	0.34	2.25	1.35	3.85	0.002	1.94	1.25	3.05	0.004	2.14	1.53	3.02	<0.001
Age	1.0	0.98	1.01	0.70	1.00	0.98	1.02	0.98	1.01	0.99	1.02	0.37	1.01	0.99	1.02	0.46
First wave^a^					0.38	0.18	0.75	0.01	0.65	0.33	1.21	0.20				
Chronic respiratory disease^b^	1.66	1.05	2.59	0.03												
Neurological symptoms^c^	1.71	1.14	2.63	0.01	40.76	8.85	724.2	<0.001	1.51	0.93	2.52	0.10	1.61	1.11	2.35	0.01
Respiratory symptoms^c^	4.44	2.03	11.68	0.001												
Gastrointestinal symptoms^c^													1.48	1.06	2.07	0.02
Vaccination^d^					1.31	0.69	2.63	0.42	0.55	0.32	0.97	0.04	0.70	0.47	1.05	0.08
Early therapy (monoclonal antibody)^e^	0.19	0.06	0.50	0.002	0.16	0.03	0.56	0.01	0.16	0.04	0.46	0.003	0.32	0.16	0.60	0.001
Corticosteroid therapy^c^					0.41	0.24	0.70	0.001								
Anticoagulant therapy^c^									1.19	0.75	1.89	0.46				
ICU admission^c^	1.22	0.76	1.94	0.40												

OR: odd ratio; CI: confident interval; LB: lower bound; UB: upper bound.

a First wave: SARS-CoV-2 infection before September 2020.

b Including asthma, chronic obstructive pulmonary disease, obstructive sleep apnoea syndrome, pulmonary hypertension, restrictive lung diseases.

c During acute infection.

d Before or after acute infection.

e Monoclonal antibodies administered within the first three days of symptoms in high risk patients (see description in the Methods section of the manuscript): bamlanivimab, bamlanivimab/etesevimab, casirivimab/imdevimab.

Early treatment with monoclonal Ab was independently associated with a lower risk of developing any of the four clinical phenotypes (REs: OR: 0.19, 95% CI: 0.06–0.50, p = 0.002; NSs: OR: 0.16, 95% CI: 0.03–0.56, p = 0.01; CPs: OR: 0.16, 95% CI: 0.04–0.46, p = 0.003; CFs: OR: 0.32, 95% CI: 0.16–0.60, p = 0.001); corticosteroid during the acute phase reduced the risk of NSs (OR: 0.41, 95% CI: 0.24–0.70, p = 0.001); and vaccination was found to reduce the risk of CPs (OR: 0.55, 95% CI: 0.32–0.97, p = 0.04) (Table 3).

Biochemical analyses and VoC did not show any correlation with clinical phenotypes. A higher proportion of patients with anti-S Ab titer below 16,000 BAU was observed in the NSs compared with patients without NSs between two and four months after the last dose of vaccination was received (46% vs 34%, p < 0.001) (Supplementary Figure S6).

Highest reduction in QoL was detected in REs and CPs (43.57 and 43.86 vs 57.32 in PCS-negative controls, p < 0.001; all comparisons in Supplementary Figure S4. Overall, accuracy estimates for the cluster analysis using as a comparison the WHO definition^3^ reached 86% balanced accuracy for NSs and 75% for CPS (Table 3).

### Severity of PCS

**Fig. 4:**
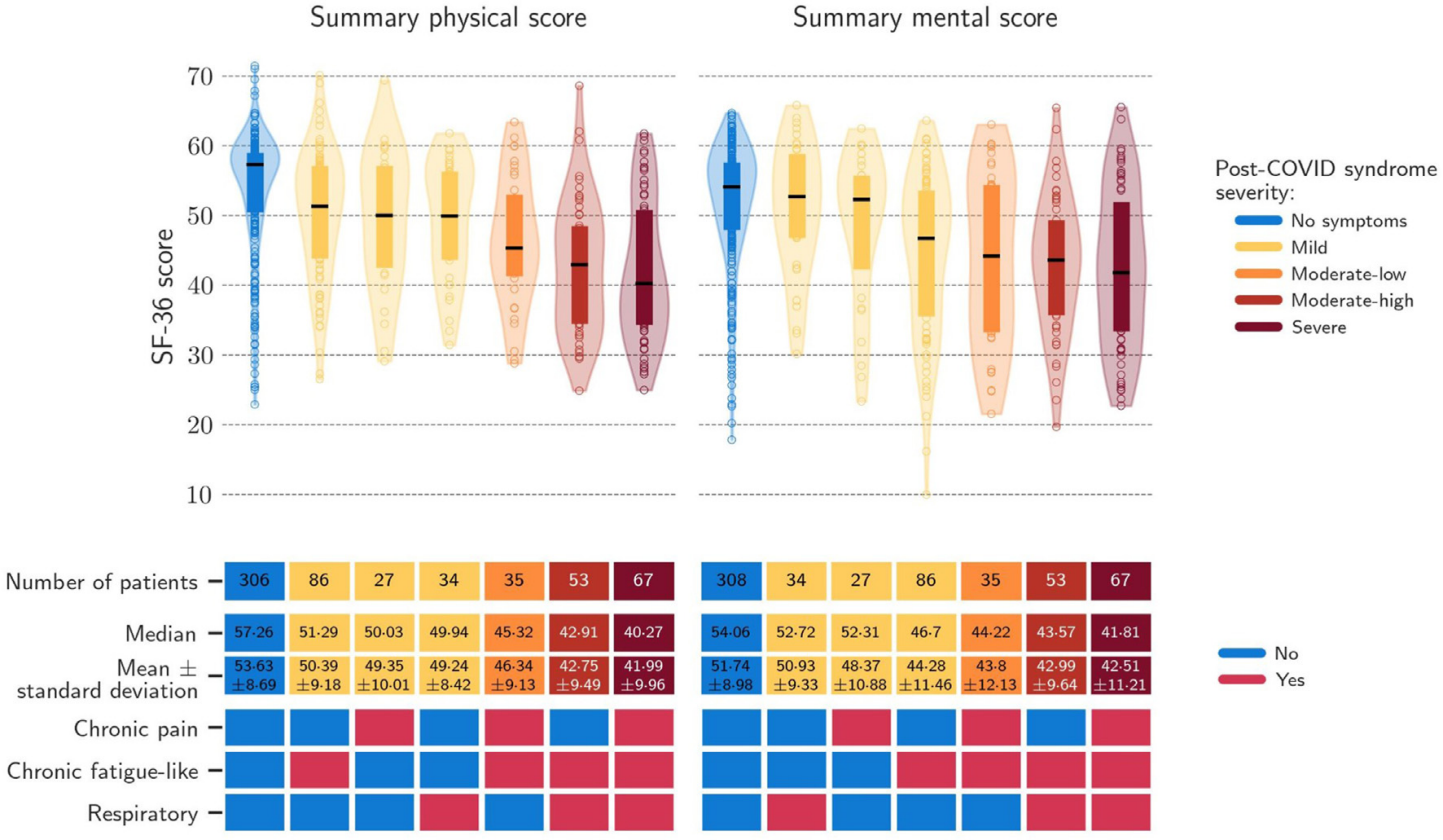
**Severity of post-COVID-19 syndrome by clinical phenotypes and quality of life measured with SF-36 questionnaire.** Chronic fatigue-like syndrome: fatigue, headache, and memory loss; respiratory syndrome: cough and dyspnoea; chronic pain: arthralgia and myalgia; and neurosensorial syndrome: altered taste and smell; SF-36: short form health survey 36.

To define severity of PCS, we analysed the impact of each of the four clinical phenotypes and their combinations on QoL at month-12 of follow-up (Fig. 4 and Supplementary Figure S7). Occurrence of three clusters at the same time (REs, NSs, and CPs) had the highest impact on QoL (41.99 ± 9.96 vs 53.63 ± 8.69 in patients with no symptoms) and was therefore considered as severe PCS. By logistic regression, being female (OR: 3.38, 95% CI: 1.81–6.53, p < 0.001), suffering from gastrointestinal symptoms (OR: 1.90, 95% CI: 1.05–3.44, p = 0.03) and/or renal complication (OR: 5.04, 95% CI: 1.76–13.91, p = 0.002) during the acute infection, independently increased the risk for severe PCS. Vaccinated patients (OR: 0.42, 95% CI: 0.22–0.81, p = 0.01) and those early treated with monoclonal Ab (OR: 0.10, 95% CI: 0.01–0.50, p = 0.03) were less likely to develop severe PCS (Table 4). The balanced accuracy of the model was 69%.

**Table 4:** Multivariable analysis of factors associated severe post-COVID-19 syndrome defined as three clusters of clinical phenotypes persistent at 12-month of SARS-CoV-2 diagnosis.

Variable	Severe post-COVID-19 syndrome (N = 501)			
	OR	95% CI		p-value
		LB	UB	
Female sex	3.38	1.81	6.53	**<0.001**
Age	1.01	0.99	1.03	0.57
Gastrointestinal symptoms^a^	1.90	1.05	3.44	**0.03**
Renal events^a^	5.04	1.76	13.91	**0.002**
Vaccination^b^	0.42	0.22	0.81	**0.01**
Early therapy (monoclonal antibody)^c^	0.10	0.01	0.50	**0.05**
Anti-viral therapy^d^	0.80	0.34	1.77	0.60
Oxygen therapy^a^	1.88	0.96	3.78	0.07

OR: odd ratio; CI: confident interval; LB: lower bound; UB: upper bound.

The significant associations denoted with a bold font refer to p-values <0.05.

a During the acute infection.

b Before or after acute infection.

c Monoclonal antibodies administered within the first three days of symptoms in high risk patients (see description in the Methods section of the manuscript): bamlanivimab, bamlanivimab/etesevimab, casirivimab/imdevimab.

d It does not include (being not available at the time of SARS-CoV-2 diagnosis of this cohort) nirmatrelvir/ritonavir and molnupinavir; it includes ribavirin, darunavir, lopinavir/ritonavir, interferon alfa, interferon beta, neuraminidase inhibitors, favipiravir, remdesivir, camostat, atazanavir.

## Discussion

Given the global spread of SARS-CoV-2 infection worldwide, we expect an increasing demand for long-term follow-up and support, affecting productivity, public health, and society.^19^ Understanding this multidimensional clinical condition is key to implementing effective preventive measures and most importantly to inform the currently empty pipeline for new treatments. The ORCHESTRA project focused on a prospective long-term data collection of SARS-CoV-2 in- and out-patients up to one year from diagnosis of acute infection, assessing clinical presentation, VoC, serological response, biochemical parameters, and QoL in order to improve accuracy of the PCS definition. Our data confirm that PCS cannot be considered a unique clinical entity, but that it can be differentiated in clinical phenotypes which are likely to have different mechanisms of pathogenesis and therefore different associated factors.^9^^,^^10^ Our results also show that the impact of the four identified clinical phenotypes on QoL is different and the assessment of risk factors, based only on nonspecific single symptom, could severely undermine the correct identification of risk and preventive factors. Comparing the analysis of risk factors between WHO definition of PCS^3^ and the newly identified clinical phenotypes, we showed that some risk factors were not previously identified (e.g., gastrointestinal symptoms at SARS-CoV-2 diagnosis) while others (e.g., neurological symptoms) seem to be strongly linked with specific clinical phenotypes. The same applies to the assessment of factors likely to decrease the risk of developing the outcomes. The analysis identified not only novel factors associated to a decreased risk to develop PCS (e.g., corticosteroid therapy during the acute phase of COVID-19) but also showed negative associations as SARS-CoV-2 vaccination and chronic fatigue syndrome. Using the criteria of presence of at least one symptom to define the PCS would underestimate the burden of disease in severe cases, thereby limiting the development of specific intensive and multidisciplinary management protocols for these patients.

Although a few studies showed that the PCS includes different clusters of symptom,^20^ risk and preventive factors by cluster have been not yet identified in a large, multicenter prospective cohort. The ORCHESTRA data support that several patient-related factors and acute infection features are associated with a higher probability of developing PCS and show that clinical phenotypes have different impact on QoL. The higher discrimination power achieved by the cluster analysis compared to the conventional logistic regression applying the WHO definition of PCS, confirms the accuracy of results, especially for the neurosensorial and chronic pain phenotypes. By combining clinical phenotypes and QoL we also propose the first definition, to the best of our knowledge, of severe PCS as a combination of three symptom clusters with highest impact on QoL, in particular on the physical component. Female sex, gastrointestinal symptoms and renal complications during the acute infection were associated with a higher risk of developing severe PCS, while vaccination and early treatment for SARS-CoV-2 were inversely associated to the outcome.

Consistently with the previous reports,^21^ our cohort showed a higher proportion of post-COVID-19 syndrome among females of reproductive age. Women elicit a stronger humoral and cellular immune response compared to men. Sex hormones and genetic factors have been proposed as underlying mechanisms for these differences,^22^ and could also explain the female prevalence of post-COVID-19 syndrome in adults. Pre-existing chronic pulmonary diseases increase the risk of the respiratory clinical phenotype of PCS.^23^ Several mechanisms could increase SARS-CoV-2 infection susceptibility in chronic pulmonary diseases, including apoptosis and epithelial damage in the airway.^24^ As for the risk of PCS, the coexistence of both diseases could possibly multiply their effects with substantial impact on QoL and physical activity.

Neurological symptoms are common in PCS. In a cohort study of 154,068 individuals with COVID-19, there was increased risk of neurologic sequelae with a hazard ratio of 1.42 and burden of 70.69 per 1000 individuals at 12-month follow-up post-acute infection. The risk was high also in individuals not requiring hospitalization.^25^ Most probable mechanisms may involve activation of microglia and astrocytes, disturbances in synaptic signalling of upper-layer excitatory neurons, and impaired neurogenesis.^25^ Our study substantially adds to the existing evidence suggesting that neurological symptoms during acute infection increase the risk not only, as expected, of the neurological clinical phenotype, but also the respiratory and the chronic fatigue-like clinical phenotypes, suggesting that these clinical phenotypes may share the same pathogenetic mechanisms.

Our analysis suggests that gastrointestinal symptoms at acute phase increases the risk of the chronic fatigue clinical phenotype. Alteration of microbiome, reported in patients with PCS six months after diagnosis of acute infection, may be responsible for the persistence of symptoms. It is possible to hypothesize that changes in the intestinal microbiome could increase the risk of persistent fatigue inducing a long-term pro-inflammatory status as demonstrated for neuropsychiatric, metabolic, and autoimmune diseases.^26^

A very recent systematic review analysed 16 observational studies from five countries assessing the effect of vaccination on development of PCS. Ten studies showed a significant reduction in the incidence with an effect increased by number of dosages. Major limitation of included studies was the lack of adjustment for potential confounders.^27^ Analysis of our cohort shows that vaccination (any number of doses, and either before or after infection) independently reduces the risk of the chronic pain clinical phenotype of the PCS. The benefit of vaccination may be explained by enhanced clearance of persistent virus or nonspecific immunomodulation, which may reduce the possible inflammatory drivers of the post-COVID-19 syndrome.^28^

The impact of treatment of moderate and severe COVID-19 on the incidence of PCS is still an open question. We observed that corticosteroids administration during the acute infection reduced the risk of developing the neurological clinical phenotype, thus suggesting a possible role of acute inflammation in the pathogenesis of post-COVID neurosensorial impairment. Early therapy with anti-SARS-CoV-2 monoclonal antibodies in high-risk patients has been shown to be effective at preventing progression to severe disease, hospitalization, and death.^29^ Our cohort provides the first evidence that early treatment with monoclonal antibodies decreases all clinical phenotypes of PCS 12 months after treatment. It is possible to hypothesize that the reduction of viral persistence in tissue and of viral mimicry (and indirectly duration of symptoms) could contribute to the reduction of risk of PCS. However, generalisability of these results should be interpreted with caution, given that the efficacy of monoclonal antibodies is strictly related to the type, virulence, and drug sensitivity of VoC.

The impact of COVID-19 on QoL has been assessed through different studies and highly heterogeneous set of tools and time points. A recent European Respiratory Society statement on long COVID follow-up, underlined a lack of consistency in the selection of instruments to measure QoL among studies.^30^ The strength of our cohort is the assessment of both in- and out-patients using the same tool at the same time points. Interestingly, being hospitalized was independently associated with a worse performance on physical activity but did not impact the mental component. QoL analysis by clinical phenotypes allowed us to stratify the PCS by severity, providing a new insight into PCS research and relevant information when developing post COVID management plans.

Limitations of our study are the high proportion of patients hospitalised vs community managed patients, reflecting that the majority of patients were enrolled from the first and second waves. However, the ORCHESTRA cohort includes also a proportion of outpatients, allowing for comparisons according to patients’ settings. Although we had a specific protocol for symptoms assessment and expert personnel prospectively assessed patients, we cannot exclude a slight overestimation of PCS, being some of the symptoms as asthenia, difficult to objectively measure it, in case the symptom was already reported before SARS-CoV-2 infection. Although early therapy of acute infection is confirmed in all analyses as a factor associated with a lower risk of PCS, treatment includes only monoclonal antibodies, and the role of oral antivirals (i.e., nirmatrelvir/ritonavir and molnupinavir), being available at later stage, could not be assessed. Worthy of note, the recommendation for using monoclonal antibodies by national and international stakeholders, was limited to patients at high risk for progression of disease so their role in preventing PCS in young population could not be analysed. The analysis of VoC in our cohort is limited, although results resembles the distribution of VoC observed in Europe during the study period. Substantial changes in virulence of new VoC could impact PCS symptoms and its incidence. Finally, it has to be noticed that large sample size may impact statistical significance, so that even small or modest effect sizes (e.g., odds ratios) appear statistically significant. We are confident, however, that our study substantially adds to the ongoing discussion on the definition of PCS. Major strengths of our study are the use of a multinational prospective cohort adopting the same protocol, the inclusion of immunological and biochemical tests, and the contemporary correlation with QoL.

In conclusion, to the best of our knowledge, this is the first attempt to propose a new clinical classification of the PCS and its severity based on clinical presentation at SARS-CoV-2 diagnosis, patients’ epidemiological and clinical characteristics, and impact of defined clinical phenotypes on QoL. Our study provides evidence that the PCS has various clinical presentations, likely driven by multiple mechanisms, and with different impact on QoL. Early identification of patients at risk of PCS, at SARS-CoV-2 diagnosis, could be used to facilitate the enrolment in new developed management protocols for follow-up in dedicated out-patient clinic just after diagnosis and drive inclusion of patients at risk for severe PCS in clinical trials for new treatment. The definition of clinical clusters could also support comparative analyses among different European and non-European cohorts and the process of data homogenisation which plays as essential role in country pandemic plans. The role of early therapy and vaccination in reducing all or some specific clinical phenotypes further support vaccination campaigns and new studies on the role of antivirals and early treatment in patients with no comorbidities are needed. There is an urgent need for new drugs to treat the sequelae of SARS-CoV-2 infection. Even if the COVID-19 pandemic comes to a close, the number of individuals suffering in the years to come from PCS, ethically does not allow us to curtail basic and clinical research in this field.

## Contributors

ET, EG, FM, AT, ZPB, JRB, GLH, MG, and CL conceptualised the study. ET, AG, MM, EG, AT, ZPB, JRB, GLH, MG, CL, and FM contributed to the methodology and the study protocol. EG, NC, PDN, AS, LDP, ZP, BTFVDG, AMF, ER, SKS, SM, JG, and MGC coordinated the study locally. ET supervised the study. LMC was involved in the project management. EG, AG, MM, NC, LDP, GDB, TO, NL, RG, SC, ZP, and ER were involved in data management. AG, RG, ILR, and JH performed the statistical analyses. AG, RG, and ILR performed data visualization. ET, EG, and AG wrote the first draft of the manuscript. EG, AG, ET, ER, PDN, LMC, AT, ZP, BTFVDG, AMF, JG, ZPB, JRB, MGC, GLH, MG, and CL reviewed the manuscript. All authors contributed to the manuscript and approved the final version of the article. All authors could access all data in the study and the corresponding author had responsibility for protecting the dataset. AG, ILR, and RG accessed and verified the data. ET and EG were responsible for the decision to submit the manuscript.

## Data sharing statement

Data will be available upon request from the corresponding author.

## Declaration of interests

AT received a grant from the Netherlands Organization for Health Research and Development (ZonMw) (grant number 10430012010023). JG received funding from ViiV Healthcare, Gilead Sciences, Theratechnologies, Astra-Zeneca, and Merck, all outside this study. MG received funding for lectures at educational events from Pfizer, Shionogi, MSD, Menarini, and Gilead, all outside this study. All other authors have nothing to declare.

## Appendix A Supplementary data

Supplementary Material

The ORCHESTRA Study Group
