# Supplementary material for: Clinical phenotypes and quality of life to define post-COVID-19 syndrome: a cluster analysis of the multinational, prospective ORCHESTRA cohort
Source: eClinicalMedicine. 2023 Jul 21;62:102107. doi: 10.1016/j.eclinm.2023.102107 (PMC10466236; doi:10.1016/j.eclinm.2023.102107)
Supplement: Supplementary Material [file mmc1.docx]

**Clinical phenotypes and quality of life to define post-COVID-19 syndrome: a cluster analysis of the multinational, prospective ORCHESTRA cohort**

Elisa Gentilotti, Anna Gorska, Adriana Tami, Roy Gusinow, Massimo Mirandola, Jesus Rodriguez-Baño, Zaira Palacios-Baena, Elisa Rossi, Jan Hasenauer, Iris Lopes-Rafegas, Elda Righi, Natascia Caroccia, Salvatore Cataudella, Zeno Pasquini, Thomas Osmo, Lidia Del Piccolo, Alessia Savoldi, Samir Kumar-Singh, Fulvia Mazzaferri, Maria Giulia Caponcello, Gerolf de Boer, Gabriel Levy Hara, Pasquale De Nardo, Surbhi Malhotra, Lorenzo Maria Canziani, Jade Ghosn, Aline-Marie Florence, Nadhem Lafhej, Bernardina T. F. van der Gun, Maddalena Giannella, Cedric Laouenan, PhD, Tacconelli Evelina and the ORCHESTRA study group

TABLE OF CONTENTS

[SECTION 1: METHODS 4](#_Toc138852460)

[Study design 4](#_Toc138852461)

[Objectives 4](#_Toc138852462)

[Inclusion criteria 4](#_Toc138852463)

[Exclusion criteria 4](#_Toc138852464)

[Enrolment and structure of the follow up 4](#_Toc138852465)

[Data collection 5](#_Toc138852466)

[Assessment of the quality of life through SF-36 questionnaire 5](#_Toc138852467)

[Principal Component Analysis (PCA) 6](#_Toc138852468)

[Figure S1 Results of the PCA analysis 7](#_Toc138852469)

[SECTION 2: DESCRIPTION OF THE COHORT AND ENROLMENT PROCEDURES 8](#_Toc138852470)

[Table S1*.* Definitions 8](#_Toc138852471)

[Table S2. Summary of the WP2 ORCHESTRA cohorts and ethic approvals 11](#_Toc138852472)

[Table S3. Epidemiological and demographic characteristics by cohort 12](#_Toc138852473)

[Table S4*.* Schedule of follow-up 13](#_Toc138852474)

[SECTION 3: ADDITIONAL RESULTS 16](#_Toc138852475)

[Table S5. Univariable analysis of features associated with the presence of at least one symptom at 12-month follow up assessment and SF-36 physical component score <50. P-values are calculated after Bonferroni correction. 16](#_Toc138852476)

[Table S6. Comparison of demographic and epidemiological characteristics between females and male patients with SARS-CoV-2 included in the cohort and followed-up to 12 months 21](#_Toc138852477)

[Table S8. Comparison of demographic and epidemiological characteristics between hospitalized and non-hospitalized patients with SARS-CoV-2 included in the cohort and followed-up to 12 months 25](#_Toc138852478)

[Figure S2. Differences in blood test parameters between patients with (red) and without (blue) at least one symptom at 6- and 12-month follow-up 28](#_Toc138852479)

[Figure S3. Density plot of SF-36 PCS and MCS scores in 1193 patients at 12-month follow-up 29](#_Toc138852480)

[Figure S4. Severity of post-COVID-19 syndrome by symptom cluster and quality of life reported by distribution of the physical and mental components of the SF-36 questionnaire at 12-month follow-up 30](#_Toc138852481)

[Figure S5. Results of the univariable analysis of factors associated with the four clinical phenotypes: respiratory, neurosensorial, chronic pain and chronic fatigue-like 31](#_Toc138852482)

[Figure S6. Differences in anti-S response between patients with and without post-COVID-19 syndrome at 12-month follow-up according to time since last vaccination against SARS-CoV-2 32](#_Toc138852483)

[Figure S7. Severity of post-COVID-19 syndrome according to clinical phenotype and quality of life reported by distribution of the physical component of the SF-36 questionnaire and subdomains (physical functioning, role limitations due to physical health, pain, and general health) at 12-month follow up 33](#_Toc138852484)

[SECTION 4: LITERATURE REVIEW 34](#_Toc138852485)

[Table S8. Literature review of systematic reviews and meta-analysis reporting on long COVID symptoms at 12 months after acute infection 34](#_Toc138852486)

[Table S9. Literature review of studies assessing the impact of post-COVID-19 syndrome on the quality of life (QoL) 37](#_Toc138852487)

[References 40](#_Toc138852488)

##

## SECTION 1: METHODS

## Study design

The ORCHESTRA WP2 includes 6 prospective cohorts (56 centers) from 5 countries (France, Italy, Netherlands, Spain, and Argentina) with SARS-CoV-2 infection laboratory confirmed between February 2020 and December 2021 and followed up at 3-, 6-, and 12-months post-acute infection. For the present study, data were extracted on 27.10.2022 from the REDCap database, to include all of the patients with available 12-months follow-up assessment by 30 June 2022, together with the information on acute infection.

The study is registered on ClinicalTrial.gov (ID: NCT05097677) and the protocol is available at institution website.

## Objectives

The primary objectives of the study were: to describe prevalence, duration, and clustering of symptoms of post-COVID-19 syndrome; to investigate preventive and risk factors for post-COVID-19 syndrome by clusters of symptoms, comorbidities, severity and treatment of acute infection (including early treatments), vaccination status, VoC, and anti-S Ab titer; and to analyse severity of post-COVID-19 syndrome by impact of clusters of symptoms on patients’ quality of life.

## Inclusion criteria

- age >14 years old;
- laboratory-confirmed SARS-CoV-2 infection;
- written informed consent.

## Exclusion criteria

- age< 14 years old;
- clinical diagnosis of SARS-CoV-2 with no available laboratory confirmation;
- no written informed consent signed.

## Enrolment and structure of the follow up

Both patients requiring hospital admission during the acute infection and outpatients could be enrolled. Patients were enrolled during the acute infection (baseline) or at any of the subsequent time points. In the first case, data about the acute infection were collected respectively, while in the second case they were retrieved retrospectively through patient’s interview and medical records. The follow up time points were the following: 3-, 6, and 12-month post-infection. Each follow up visit combines clinical and laboratory assessment, as reported in table S4. Nasal swab was performed to define the variant of concern (VOC) at baseline and repeated only in case of positive sampling after 30 days since infection diagnosis. VoC and serological analysis were performed at central laboratory of Antwerp (SAS, UNIBO, and UNIVR) or at local laboratories (COVID Home, INSERM, and UBA) using homogenised protocols.). Serological results in AU/ml were converted into BAU by multiplying with a seroconversion factor (1.0288 for Roche Elecsys Anti-SARS-CoV-2; 0.142 for Alignity_Abbott; and 0.00901 for MSD assay). For the purpose of the analysis, immunological anti-S response was classified as: <1500 BAU, 1500-5000 BAU, 5000-16000 BAU, and >16000 BAU.

## Data collection

Study data were collected and managed using REDCap electronic data capture tools hosted at CINECA. REDCap (Research Electronic Data Capture) is a secure, web-based software platform designed to support data capture for research studies, providing 1) an intuitive interface for validated data capture; 2) audit trails for tracking data manipulation and export procedures; 3) automated export procedures for seamless data downloads to common statistical packages; and 4) procedures for data integration and interoperability with external sources. The variables underwent a process of homogenization across the different cohorts and standardization according to the protocol. Since the cohorts in France (French COVID and in the Netherlands (COVID-HOME) started before the ORCHESTRA project was financed, data from these two cohorts went through a post-data collection harmonization process under the supervision of the Charité – Universitätsmedizin Berlin and transformation conducted by the Centre Informatique National de l’Enseignement Superieur (CINES).

Data collected at baseline included date of symptom onset and diagnosis, duration of symptoms, demographic characteristics, comorbidities, clinical presentation, treatment during the acute infection, hospitalization, admission to ICU, and post-acute infection complications.Early treatment was defined as monoclonal antibodies administered during the study period (bamlanivimab, bamlanivimab/etesevimab, casirivimab/imdevimab), received within the first 5 days of onset of symptoms according to national recommendations (data available only for UNIVR cohort). A symptom was associated to SARS-CoV-2 infection if newly diagnosed after acute infection or if a significant worsening in terms of severity and/or presentation of the symptom was registered after acute infection. Occurrence of new medical events, vital signs and physical examination, laboratory parameters and vaccination status were also collected at each time point (table S4). Quality checks of variables were run by CINECA and errors were reported back to the cohort’s local teams for correction. Data imputation was performed only in case of data that could be derived by known information, such as availability of the vaccination (e.g., patients infected before the start of vaccination campaigns within each country were considered as not vaccinated).

## Assessment of the quality of life through SF-36 questionnaire

The quality of life was assessed through the 36-Item Short Form Survey (SF-36). The SF-36 questionnaire is composed of 36 items categorised into 8 scales: physical functioning, role limitations due to physical health, pain, general health, vitality, social functioning, role limitations due to emotional problems, and mental health. Each of the items had from 2 to 6 levels of answers. For each scale, the items were (re)codified, transformed, and aggregated into a scale ranging from 0 to 100. In case of missing information, if the respondent has answered at least 50% of the items within the scale, the scale average was imputed into the missing items. Once the score for each of the 8 scales was computed, these were aggregated into two main components: the physical component summary (PCS) and the mental component summary (MCS) based on population-representative weights computed via PCA. The questionnaires were scored using the PRO CoRE software developed by QualityMetrics, which applies US1998 norms. Definition of suboptimal score was based on the 25^th^ percentile of the distribution for patients not reporting symptoms at the 12-month assessment.

##

## Principal Component Analysis (PCA)

Based on both frequency and completeness of available information across all the cohorts, we restricted the number of symptoms to the following nine: cough, dyspnoea, fatigue, memory loss, headache, ageusia, anosmia, myalgia and arthralgia. We utilized an unsupervised machine learning algorithm called principal component analysis (PCA) to identify groups of symptoms. These groups were later used as results in the univariable and multivariable logistic regression to examine factors associated to each of the identifiedclinical phenotype and their impact on quality of life measured with the SF-36 questionnaire. To reduce the number of symptoms, PCA was utilized as a tool for dimensionality reduction. We created new dimensions based on linear combinations of the original variables that captured the highest variation while maintaining orthogonality between any two principal components.

Figure S1 a) graph shows the percentage variance attributed to PCA components of the nine largest eigenvalues. The first three components are essential, as they have corresponding eigenvalues that are more significant than one and explain around 54% of the total variance of the dataset. Figure S1 b) presents a matrix showing the oblique-rotated loading of the first three principal components. According to this matrix,Anosmia/Ageusia and Arthralgia/Myalgia have larger loading values and contribute more to the 2nd and 3rd components, respectively. The third cluster was divided into two based on clinical significance to better understand the various symptoms associated with cough, dyspnoea, fatigue, headache, and memory loss. This resulted in four distinct clinical phenotypes: Chronic Pain (CPc), Neurosensorial (NSc), Respiratory (Res), and Chronic Fatigue (CFs).

Figure S1 Results of the PCA analysis

**
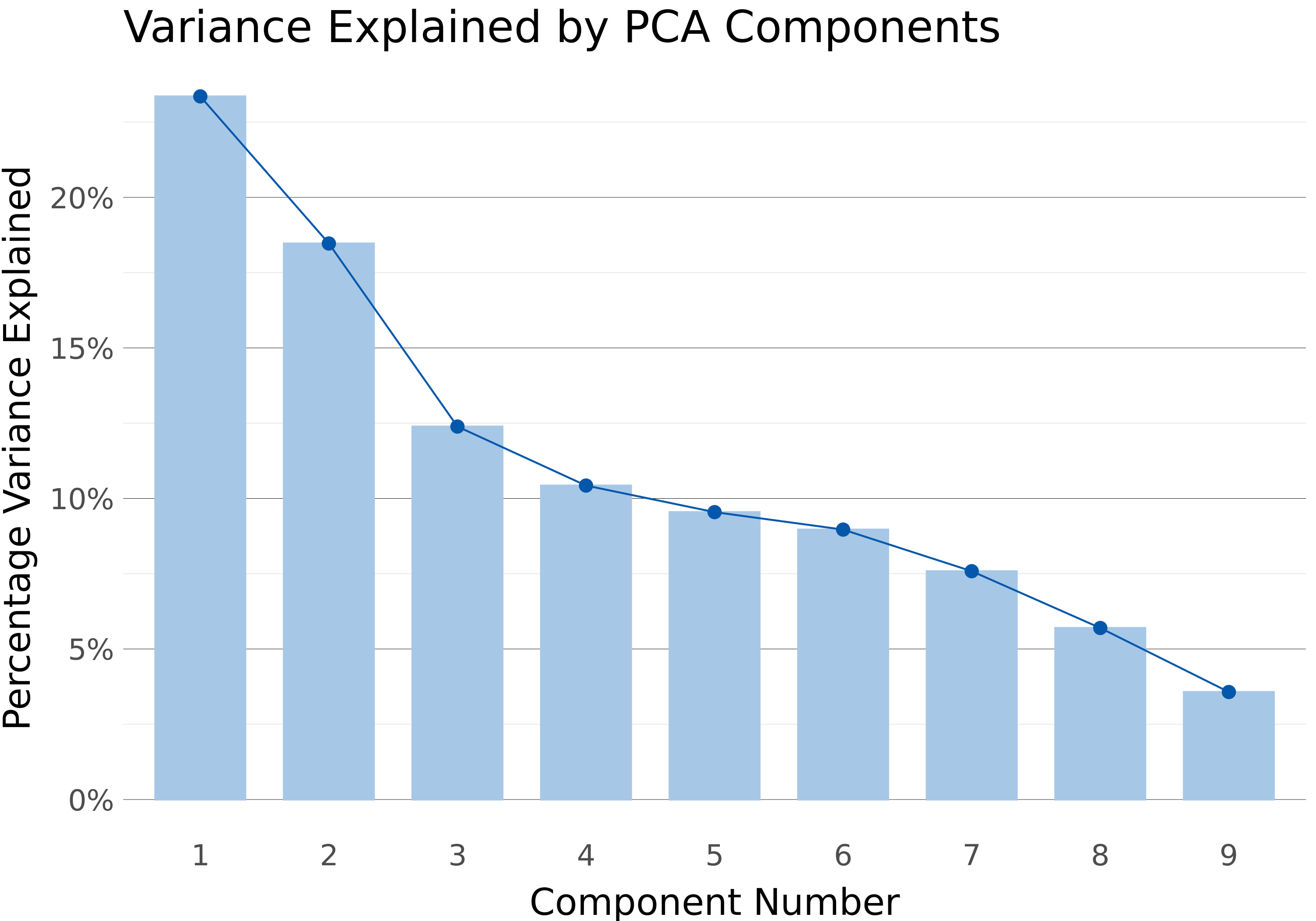
**

a) Variance Explained by PCA Components

**
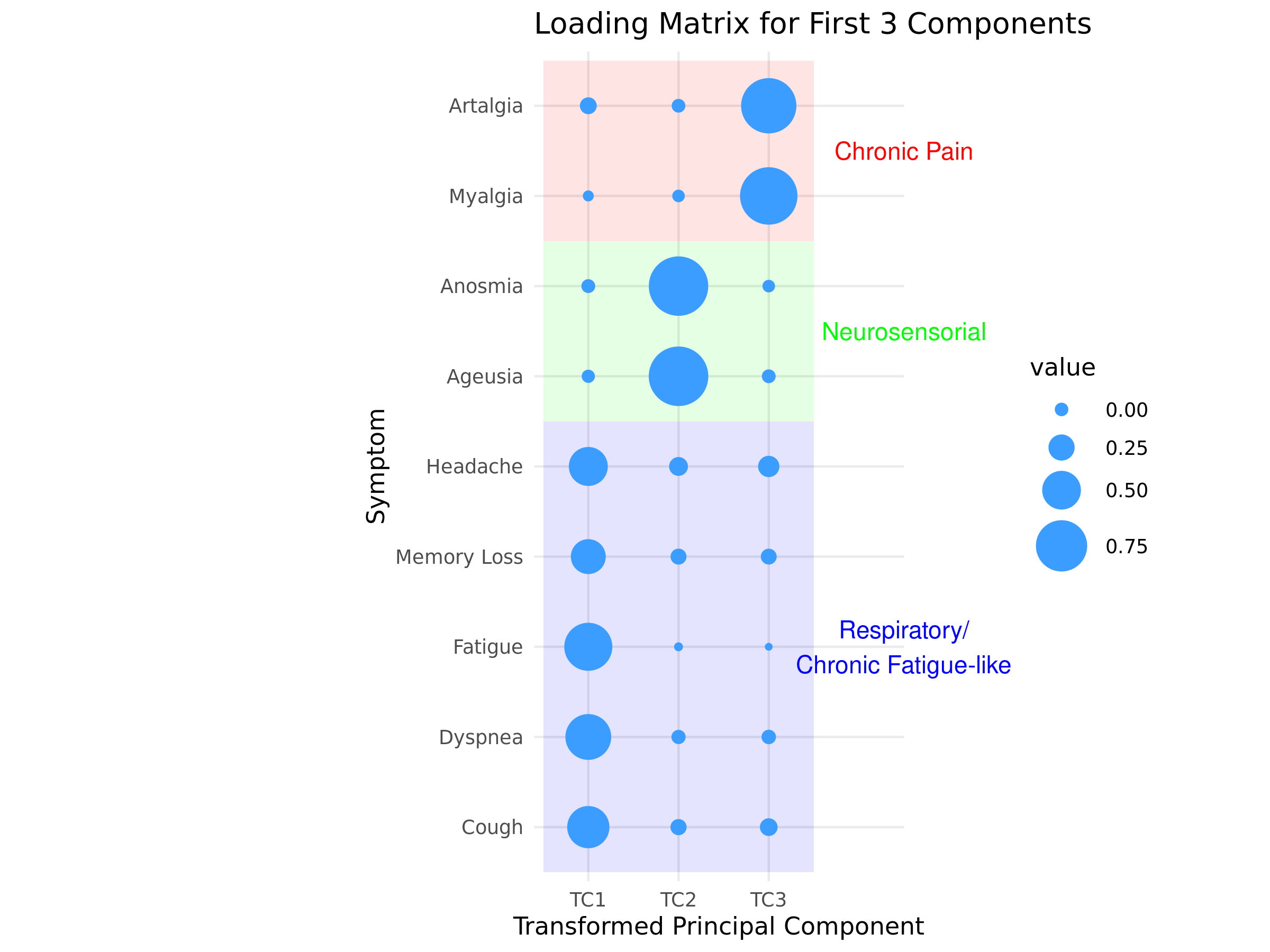
**

b) Loading Matrix Visualisation for first three components

## SECTION 2: DESCRIPTION OF THE COHORT AND ENROLMENT PROCEDURES

## Table S1*.* Definitions

|  | **Definition** |
| --- | --- |
| **Cardiovascular disease** | Hypertension  Congestive heart failure  Coronary heart disease |
| **Chronic respiratory disease** | Asthma  Chronic obstructive pulmonary disease (COPD)  Obstructive sleep apnea syndrome (OSAS)  Pulmonary hypertension  Restrictive lung disease |
| **Diabetes** | Diabetes type 1  Diabetes type 2 |
| **Renal disease** | Kidney damage for > 3 months – includes:  Primary glomerular disease (focal segmental glomerulosclerosis, IgA nephropathy)  Secondary glomerular disease (diabetes with renal complications, systemic lupus erythematosus)  Tubulointerstitial diseases (sarcoidoisis, drug-induced, urate, environmental toxins, myeloma)  Obstructive nephropathy  Vascular diseases (Atherosclerosis, hypertension, ischemia, cholesterol emboli, systemic vasculitis, thrombotic microangiopathy, systemic sclerosis)  Cystic and congenital diseases (Polycystic kidney disease, Alport's syndrome, Fabry's disease) |
| **Liver diseases** | Chronic liver disease other than cancer includes:  Alcohol induced liver disease  Non-alcoholic fatty liver disease (NAFLD)  Non-alcoholic steatohepatitis (NASH)  Autoimmune Hepatitis (AIH)  Primary Biliary Cirrhosis (PBC)  Hereditary Hemochromatosis  Wilsons' Disease |
| **Active cancer** | Solid tumor  Haematological malignancy |
| **Transplant recipients** | Solid organ  Bone marrow |
| **Autoimmune diseases** | Rheumatic disease  Inflammatory bowel disease (ulcerative colitis, Crohn's disease)  Psoriatic rheumatism  Autoimmune hepatitis  Psoriasis  Atopic dermatitis  Chronic urticaria  Multiple sclerosis  Inflammatory myopathy  Systemic lupus erythematosus  Systemic scleroderma  Sjögren’s syndrome  Behcet's syndrome  Atrophic polychondritis  Antiphospholipid syndrome  Takayasu arteritis  Horton disease  Knotty periarthritis  Kawasaki's disease  Microscopic polyangiitis  Wegener's disease  Churg-Strauss syndrome  Rheumatoid purpura  Buerger's disease  Cryoglobulinemia  Sarcoidosis |
| **Steroid therapy** | Dexamethasone  Prednisone  Methylprednisolone |
| **Anticoagulant therapy** | Unfractioned heparin  Low molecular weight heparin  Fondaparinux  Vitamin K antagonist (ex. Warfarin)  Direct oral anticoagulants (ex. dabigatran, rivaroxaban, apixaban, edoxaban, betrixaban) |
| **Antiviral therapy** | Ribavirin  Lopinavir/ritonavir  Interferon alpha  Interferon beta  Neuraminidase inhibitors  Favipiravir  Remdesivir  Camostat  Atazanavir  Darunavir |
| **Monoclonal antibodies therapy** | Bamlanivimab  Bamlanivimab plus etesevimab  Casirivimab plus imdevimab |
| **Immunomodulator therapy** | Tocilizumab  Sarilumab  Canakinumab  Siltuximab  Acalabrutinib  Ruxolitinib  Adalimumab  Etanercept  Baricitinib  Anakinra  Emapalumab  Cyclosporin A  Tacrolimus  Sirolimus  Everolimus |
| **General symptoms** | Fever  Fatigue  Myalgia  Arthralgia  Headache  Conjunctivitis  Lymphadenopathy  Anorexia  Skin rash  Haemorrhage |
| **Respiratory symptoms during acute infection** | Cough  Dyspnoea  Sore throat  Nasal congestion  Rhinorrhoea  Chest pain  Chest retraction  Wheezing |
| **Gastrointestinal symptoms during acute infection** | Abdominal pain  Diarrhoea  Nausea  Vomiting |
| **Neurological symptoms during acute infection** | Ageusia  Anosmia  Syncopal episodes  Confusion  Memory loss  Aphasia  Anomia  Seizures  Inability to walk |
| **Pulmonary complications** | Pulmonary aspergillosis  Pneumothorax  Pleural effusion  Cryptogenic organizing pneumonia |
| **Cardiac complications** | Congestive heart failure  Cardiac arrhythmia  Myocarditis  Pericarditis  Cardiac ischemia  Cardiac arrest |
| **Embolic complications** | Pulmonary embolism  Deep vein thrombosis  Other thromboembolic event  Disseminated intravascular coagulation |
| **Neurological complications** | Meningitis  Encephalitis  Seizure  Stroke or cerebrovascular accident |
| **Renal complications** | Acute renal injury  Acute renal failure |
| **Gastrointestinal complications** | Gastrointestinal hemorrhage  Pancreatitis  Acute liver dysfunction |
| **Serology assessment** | Serological results in AU/ml were converted into BAU by multiplying with a seroconversion factor:   - 1.0288 for Roche Elecsys Anti-SARS-CoV-2; - 0.142 for Alignity_Abbott; and - 0.00901 for MSD assay   For the purpose of the analysis, immunological anti-S response was classified as:   - <1500 BAU, - 1500-5000 BAU, - 5000-16000 BAU, and - >16000 BAU |

## Table S2. Summary of the WP2 ORCHESTRA cohorts and ethic approvals

| **Cohort** | **Ethical committee approval and enrollment start** |
| --- | --- |
| **ORCHESTRA – University of Verona, *Italy*** | 13^th^ April 2021 |
| **ORCHESTRA – University of Bologna UNIBO, *Italy*** | 3^rd^ June 2021 |
| **ORCHESTRA- French Covid, Institut National de la Santé et de la Recherche Médicale, *France*** | 7^th^ February 2020 |
| **ORCHESTRA - Andalusian Health Service, *Spain*** | 19^th^ March 2020 |
| ***ORCHESTRA -* COVID-HOME, University Medical Center Groningen, the *Netherlands*** | 28^th^ May 2021 |
| **ORCHESTRA - Universidad de Buenos Aires, *Argentina*** | 8^th^ July 2021 |

## Table S3. Epidemiological and demographic characteristics by cohort

| **Country** | **All**  **n (%)** | **France**  **n (%)** | **Italy (Verona)**  **n (%)** | **Italy (Bologna)**  **n (%)** | **Spain**  **n (%)** | **Argentina**  **n (%)** | **The Netherlands**  **n (%)** |
| --- | --- | --- | --- | --- | --- | --- | --- |
| **Population (in-/outpatients)** | Mixed | Inpatients | Mixed | Mixed | Inpatients | Outpatients | Outpatients |
| **Number of patients** | 1796 | 929 | 553 | 113 | 53 | 14 | 134 |
| **Age, mean**±SD | 57·19 ± 14·95 | 60·75 ± 13·62 | 55·21 ± 14·62 | 57·27 ± 14·43 | 54·4 ± 13·24 | 40·64 ± 12·47 | 43·54 ± 15·58 |
| **BMI, mean**±SD | 27·64 ± 5·02 | 28·34 ± 5·01 | 28·05 ± 5·01 | 29·63 ± 4·8 | 25·65 ± 5·6 | -- | 25·06 ± 4·09 |
| **Female** | 779 (43·4) | 335 (36·06) | 302 (54·61) | 42 (37·17) | 22 (41·51) | 1 (7·14) | 77 (57·89) |
| **Smoker** | 112 (7·06) | 43 (5·23) | 41 (7·88) | 4 (8·89) | 13 (24·53) | 3 (21·43) | 8 (6·06) |
| **Vaccinated before acute infection** | 263 (14·82) | 11 (1·18) | 107 (19·38) | 21 (18·75) | 11 (20·75) | 5 (35·71) | 108 (93·91) |
| **Vaccinated after acute infection** | 1082 (73·81) | 321 (50·87) | 487 (88·07) | 101 (94·39) | 52 (98·11) | 13 (92·86) | 108 (100·0) |
| **Diabetes^a^** | 221 (12·51) | 152 (16·83) | 42 (7·61) | 15 (13·27) | 8 (15·09) | 0 (0·0) | 4 (3·05) |
| **Autoinflammatory disease^a^** | 109 (6·07) | 71 (7·64) | 33 (5·97) | 4 (3·54) | 1 (1·89) | 0 (0·0) | 0 (0·0) |
| **Cardiovascular disease^a^** | 714 (41·37) | 415 (47·98) | 222 (40·22) | 48 (42·48) | 24 (45·28) | 2 (14·29) | 3 (2·33) |
| **Chronic liver disease^a^** | 52 (2·93) | 30 (3·24) | 12 (2·18) | 6 (5·36) | 3 (5·66) | 1 (7·14) | 0 (0·0) |
| **Chronic kidney disease^a^** | 90 (5·1) | 72 (7·79) | 15 (2·76) | 2 (1·8) | 1 (1·89) | 0 (0·0) | 0 (0·0) |
| **General symptoms^a^** | 1030 (57·35) | 607 (65·34) | 287 (51·9) | 44 (38·94) | 36 (67·92) | 1 (7·14) | 55 (41·04) |
| **Respiratory symptoms^a^** | 502 (28·06) | 339 (36·69) | 91 (16·49) | 14 (12·5) | 26 (49·06) | 0 (0·0) | 32 (23·88) |
| **Neurosensorial symptoms^a^** | 197 (11·05) | 78 (8·5) | 79 (14·31) | 1 (0·9) | 4 (7·55) | 0 (0·0) | 35 (26·12) |
| **Gastrointestinal symptoms^a^** | 39 (4·55) | No data | 12 (2·19) | 2 (1·82) | 3 (5·77) | 0 (0·0) | 22 (16·42) |

^a^See table S1 for definitions; SD: standard deviation

## Table S4*.* Schedule of follow-up

|  | **COVID-19**  **(2 weeks^1^**  **± 2 weeks)** | **3 months^1^**  **± 1 month** | **6 months^1^**  **± 1 month** | **12 months^1^**  **± 1 month** | **18 months^1^**  **± 2 months** |
| --- | --- | --- | --- | --- | --- |
| **Screening/baseline** |  |  |  |  |  |
| Inclusion criteria^1^ |  |  |  |  |  |
| Demographics^2^ |  |  |  |  |  |
| Healthcare setting^3^ |  |  |  |  |  |
| Length of hospital stay, days |  |  |  |  |  |
| ICU admission |  |  |  |  |  |
| Medical history^4^ |  |  |  |  |  |
| **Treatment** |  |  |  |  |  |
| Comorbidity management^5^ | X | X | X | X | X |
| Anti-COVID therapy^6^ | X |  |  |  |  |
| Antibiotic therapy^7^ |  |  |  |  |  |
| Oxygen therapy^8^ | X | X* | X* | X* | X* |
| SARS-CoV-2 vaccination^9^ | X | X | X | X | X |
| **Clinical assessment** |  |  |  |  |  |
| Relevant medical new events^10^ | X | X | X | X | X |
| COVID-19 symptom^11^onset | X |  |  |  |  |
| COVID-19 symptom end | X | X* | X* | X* | X* |
| COVID severity^12^ | X |  |  |  |  |
| SOFA score | X |  |  |  |  |
| Vital signs^13^ | X | X | X | X | X |
| Physical examination^14^ | X | X | X | X | X |
| 12-lead electrocardiography | X | X | X | X | X |
| 6-minute walking test | X | X | X | X | X |
| DLCO (diffusing capacity for carbon monoxide) | X | X | X | X | X |
| Pulmonary function test^15^ | X | X | X | X | X |
| **Questionnaires** |  |  |  |  |  |
| Functional status^16^ | X | X | X | X | X |
| Respiratory impairment^17^ | X | X | X | X | X |
| Mental health^18^ | X | X | X | X | X |
| Perceived risk of re-infection/admission/re-admission^19^ | X | X | X | X | X |
| Adherence to main preventative non-pharmacological measures^20^ | X | X | X | X | X |
| SARS-CoV-2 vaccination: acceptance/non-acceptance and reasons^21^ | X | X | X | X | X |
| **Imaging** |  |  |  |  |  |
| Lung ultrasound | X | X | X* | X | X* |
| X-ray | X | X | X* | X* | X* |
| High-resolution CT scan | X | X | X* | X* | X* |
| Cardiac ultrasound | X | X | X* | X | X* |
| Cardiac MRI^22^ | X^21^ | X^21^ | X* | X^21^ | X* |
| **Biochemistry** |  |  |  |  |  |
| Blood tests^23^ | X | X | X* | X | X* |
| Arterial blood gas test (pO_2_/pCO_2_/pH) | X | X | X* | X* | X* |
| Urine tests^24^ | X | X | X* | X | X* |
| **Immunology** |  |  |  |  |  |
| N-IgG | X | X | X | X | X |
| N-IgM | X | X | X | X | X |
| N-IgA | X | X | X | X | X |
| S-IgG | X | X | X | X | X |
| S-IgM | X | X | X | X | X |
| S-IgA | X | X | X | X | X |
| **Microbiological tests** |  |  |  |  |  |
| SARS-CoV-2 molecular test in nasopharyngeal swab or tracheal aspirate or bronchoalveolar lavage to detect | X | X* | X* | X* | X* |
| **Adjunctive variables for specific fragile populations** | | | | | |
| **HIV** |  |  |  |  |  |
| HIV-infection status^25^ | X | X | X | X | X |
| HIV-Infection therapy^26^ | X | X | X | X | X |
| Assessment of adherence to follow-up visits and antiretroviral therapy | X | X | X | X | X |
| **Elderly** |  |  |  |  |  |
| Cognitive status^27^ | X | X | X | X | X |
| **Pregnant women/new mother** |  |  |  |  |  |
| History of positive SARS-CoV-2 molecular test on amniotic fluid or breast milk^28^ |  |  |  |  |  |
| History of detection of microthrombotic disease on placenta tissue or umbelical cord tissue |  |  |  |  |  |
| **Children** |  |  |  |  |  |
| History of positive SARS-CoV-2 molecular test on amniotic fluid or breast milk^29^ |  |  |  |  |  |
| Biometric paramethers^30^ | X | X | X | X | X |
| **Transplant** |  |  |  |  |  |
| Transplant general information^31^ |  |  |  |  |  |
| Graft function^32^ | X | X | X | X | X |
| Immunosuppressive regimen^33^ | X | X | X | X | X |
| **Onco-haematology** |  |  |  |  |  |
| Assessment of adherence to oncologic follow-up visits and therapy | X | X | X | X | X |
| Assessment of progression of the disease and relapse | X | X | X | X | X |
| Assessment of adverse events^34^ | X | X | X | X | X |

Modular data capture according to level of commitment (level I, level II, level III).

| Level I | Assessments in level I are mandatory |
| --- | --- |
| Level II | Customized according to the feasibility of each cohort |

* Reassessed only if outside the normal ranges at the previous assessment or if clinically indicated

^1^Day 0: first positive SARS-CoV-2 test. Inclusion criteria for enrollment: in- and out-patients aged >14 years old with a laboratory-confirmed SARS-CoV-2 infection who signed written informed consent. Exclusion criteria; age below 14 years, no written informed consent signed and no SARS-CoV-2 test performed;^2^Demographics: age (years), sex, ethnic group (African, Asian, European, Latin America...), education (no formal education, lower than college, college or higher), cigarette smoking (never-smoker, former smoker, current smoker), usual residence (home, long-term care facility, public dormitory, prison, homeless), current occupation (student, unemployed with no benefits, unemployed with benefits, employed, self-employed, informal worker); ^3^Healthcare setting: (a) outpatient (b) non-intensive care unit (c) intensive care unit; ^4^Medical history: cardiovascular diseases (hypertension, coronary artery disease, congestive heart failure), diabetes (without insulin, with insulin), chronic respiratory disease (asthma, chronic obstructive pulmonary disease, obstructive sleep apnoea, restrictive lung disease, pulmonary hypertension), kidney disease (chronic with/without dialysis), liver disease other than cancer (HBV/HCV/HDV chronic viral hepatitis, other chronic disease, cirrhosis), metabolic disease, immunosuppressive conditions (solid organ transplant recipient, auto-immune diseases), cancer (solid cancer, haematological malignancies, type of primitive cancer/haematological malignancies, presence of metastases, if ongoing chemotherapy), mental or neurological disorders (psychiatric illness, anxiety disorder, mood disorder, psychotic disorder, Alzheimer disease, dementia other than Alzheimer, Parkinson’s disease, myasthenia gravis, epilepsy, stroke (with/without residual deficits, neuromuscular disease, multiple sclerosis), muscular dystrophy, amyotrophic lateral sclerosis); TB co-infection; other opportunistic co-infection (specify) for HIV population; ^5^Comorbidity management: drug name and dose (to include only treatments taken regularly); ^6^Anti-COVID therapy: drug name, maintenance dose, and duration; ^7^Antibiotic therapy: drug name, dose, duration, and type of treated infection; ^8^Oxygen therapy: nasal prongs, face mask, face mask with reservoir, high-flow nasal cannula, non-invasive ventilation, mechanical ventilation; numbers of O_2_ (L/min) provided (maximum reached) and fraction of inspired O_2_ (FiO_2_) provided (maximum reached); ^9^SARS-CoV-2 vaccination: vaccine name, date of administration; ^10^Relevant new medical events or worsening of previous conditions, including deep venous thrombosis, pulmonary embolism, infections (including a new SARS-CoV-2-infection during follow-up), malignancies (type of cancer, overall stage); ^11^Symptoms: abdominal pain, ageusia/dysgeusia, anosmia, balance impairment, behaviour disorder, chest pain or chest tightness, confusion, cough, delirium, diarrhoea, disrupted sleep, dizziness, dyspnoea, fatigue, fever (including low-grade fever), headache, hypothermia, impaired cognitive status, lethargy, loss of appetite, mood affective disorder, myalgia, nausea/vomiting, palpitation, phlegm, runny nose, sore throat, stuffed nose, syncope, wheeze; ^12^WHO Clinical Progression Scale; ^13^Vital signs: dead/alive, blood pressure, body temperature, heart rate, respiratory rate, peripheral oxygen saturation; ^14^Physical examination: BMI, abdominal examination, pulmonary examination, cardiac examination, neurological examination, peripheral vascular examination; ^15^Pulmonary function test: FEV_1_, FVC, FEV_1_/FVC, TLC, FRC, RV; ^16^Questionnaires to address the functional status: Post-COVID-19 Functional Status (PCFS) Scale, Global Physical Activity, Questionnaire (GPAQ), Barthel Index, Medical Outcome Study Short Form (MOS SF)-36 Score, EuroQol five-dimension five-level (EQ-5D-5L) questionnaire, Clinical Frailty Scale (CFS), Basic Activity of Daily Living (BADL); ^17^Questionnaires to address the respiratory impairment: Saint George Respiratory Questionnaire (SGRQ), Transition Dyspnoea Index (TDI), mMRC (Modified Medical Research Council) Dyspnea Scale; ^18^Questionnaires to address the mental health: Hospital Anxiety and Depression Scale (HADS), Kessler Psychological Distress Scale (K10), Impact of Event Scale – Revised (IES-R); ^19^Perceived risk of re-infection on a scale 0-10 (no risk- very high risk); perceived risk of admission/re-admission on a scale 0-10 (no risk- very high risk); ^20^Frequency mask-wearing (type of mask); frequency hand washing; respect of social distance; avoidance of social gathering; ^21^Was the vaccine accepted? Why not accepted (lack of trust in efficacy and/or safety; not useful in the specific case; prefer someone else gets it before me); ^22^Cardiac MRI only if abnormal cardiac ultrasound; ^23^Blood tests: haemoglobin, white blood cell count, lymphocyte count, neutrophil count, platelets, sodium, potassium, creatinine, glucose, haemoglobin A1c, bilirubin, alanine aminotransferase, aspartate aminotransferase, gamma glutamyl transpeptidase, albumin, lactate dehydrogenase, ferritin, creatine kinase, fibrinogen, INR, partial thromboplastin time, D-dimer, NT-pro-BNP, troponin, C-reactive protein (CRP), procalcitonin, venous lactate; ^24^Urine tests: pH, concentration, protein, glucose, red blood, white blood cell count; ^25^CD4 lymphocyte count; HIV-viral load; AIDS status; ^26^HIV-therapy: drug name and dose (only ongoing treatment); previous switch to other regimens for virological failure; ^27^General nursing home; residential home; specialized LTCFs; mixed LTCFs, other LTCFs; overall number of beds; ownership of the facility: public, for profit, not for profit; ^28^Questionnaires to address cognitive status: Cognitive Failure Questionnaire (CFQ), Mini-Mental State Examination, Clinical Dementia rating Scale; ^29^Results of SARS-CoV-2 molecular test on amniotic fluid; ^30^Weight, height/length, cranial circumference, BMI; ^31^Type of transplant (hearth, lung, kidney, liver, pancreas); single-combined; year of transplantation; ^32^Graft function: good, impaired, failure, rejection acute-chronic, recurrence of underlying disease, other; ^33^Immunosuppressive regimen: drug name and dose; ^34^According to Common Terminology Criteria for Adverse Events (CTCAE).

## SECTION 3: ADDITIONAL RESULTS

## Table S5. Univariable analysis of features associated with the presence of at least one symptom at 12-month follow up assessment and SF-36 physical component score <50. P-values are calculated after Bonferroni correction.

| **Variable** | | **Total** | **PCS** | **%** | **Crude OR** | **95% CI** | | ***p-*value** | **Total** | **SF-36**  **PCS<50** | **%** | **Crude OR** | **95% CI** | | **p-value** |
| --- | --- | --- | --- | --- | --- | --- | --- | --- | --- | --- | --- | --- | --- | --- | --- |
|  |  |  |  |  |  | **lower** | **upper** |  |  |  |  |  | **lower** | **upper** |  |
|  | ***Demographic and clinical characteristics*** | | | | | | | | | | | | | | |
| **Sex** | |  |  |  |  |  |  |  |  |  |  |  |  |  |  |
| Male | | 1016 | 536 | 52·8 | 1 |  |  |  | 646 | 254 | 39·3 | 1 |  |  |  |
| Female | | 779 | 493 | 63·3 | 1·543 | 1·28 | 1·87 | **<0·001** | 547 | 272 | 49·7 | 1·53 | 1·21 | 1·92 | **<0·001** |
| **Age groups (years old)** | |  |  |  |  |  |  |  |  |  |  |  |  |  |  |
| 15-30 | | 98 | 50 | 51·0 | 1 |  |  |  | 67 | 15 | 22·4 | 1 |  |  |  |
| 31-40 | | 150 | 83 | 55·3 | 1·19 | 0·71 | 1·99 | 0·51 | 104 | 29 | 27·9 | 1·33 | 0·66 | 2·80 | 0·43 |
| 41-60 | | 774 | 456 | 58·9 | 1·38 | 0·90 | 2·10 | 0·14 | 553 | 235 | 42·5 | 2·54 | 1·43 | 4·79 | **0·001** |
| 61-80 | | 689 | 403 | 58·5 | 1·35 | 0·88 | 2·07 | 0·17 | 426 | 214 | 50·2 | 3·47 | 1·93 | 6·58 | **<0·001** |
| >80 | | 83 | 38 | 45·8 | 0·81 | 0·45 | 1·46 | 0·49 | 43 | 34 | 79·1 | 12·57 | 5·12 | 33·89 | **<0·001** |
| **Smoking status** | |  |  |  |  |  |  |  |  |  |  |  |  |  |  |
| Non-smoker | | 1040 | 600 | 57·7 | 1 |  |  |  | 685 | 299 | 43·6 | 1 |  |  |  |
| Former smoker | | 434 | 248 | 57·1 | 0·98 | 0·78 | 1·23 | 0·85 | 272 | 128 | 47·1 | 1·15 | 0·87 | 1·52 | 0·34 |
| Smoker | | 112 | 70 | 62·5 | 1·22 | 0·82 | 1·84 | 0·33 | 80 | 33 | 41·3 | 0·91 | 0·56 | 1·45 | 0·69 |
| **Body Mass Index** | |  |  |  |  |  |  |  |  |  |  |  |  |  |  |
| <30 | | 263 | 133 | 50·6 | 1 |  |  |  | 146 | 75 | 51·4 | 1 |  |  |  |
| ≥30 | | 138 | 85 | 61·6 | 1·57 | 1·03 | 2·39 | 0·04 | 73 | 42 | 57·5 | 1·28 | 0·73 | 2·27 | 0·39 |
| **Cardiovascular disease^a^** | |  |  |  |  |  |  |  |  |  |  |  |  |  |  |
| No | | 1012 | 569 | 56·2 | 1 |  |  |  | 714 | 261 | 36·6 | 1 |  |  |  |
| Yes | | 710 | 410 | 57·7 | 1·06 | 0·88 | 1·29 | 0·53 | 434 | 238 | 54·8 | 2·11 | 1·65 | 2·69 | **<0·001** |
| **Cardiovascular disease classification** | |  |  |  |  |  |  |  |  |  |  |  |  |  |  |
| None | | 1012 | 569 | 56·2 | 1 |  |  |  | 714 | 261 | 36·6 | 1 |  |  |  |
| Congestive heart failure | | 9 | 5 | 55·6 | 0·97 | 0·25 | 4·10 | 0·96 | 7 | 6 | 85·7 | 9·30 | 1·52 | 241·61 | **0·01** |
| Coronary heart disease | | 175 | 108 | 61·7 | 1·25 | 0·90 | 1·75 | 0·18 | 94 | 57 | 60·6 | 2·67 | 1·72 | 4·18 | **<0·001** |
| Hypertension | | 468 | 272 | 58·1 | 1·08 | 0·87 | 1·35 | 0·50 | 293 | 157 | 53·6 | 2·00 | 1·52 | 2·64 | **<0·001** |
| Other | | 58 | 25 | 43·1 | 0·59 | 0·34 | 1·01 | 0·05 | 40 | 18 | 45·0 | 1·42 | 0·74 | 2·71 | 0·29 |
| **Diabetes^a^** | |  |  |  |  |  |  |  |  |  |  |  |  |  |  |
| No | | 1545 | 883 | 57·2 | 1 |  |  |  | 1051 | 434 | 41·3 | 1 |  |  |  |
| Yes | | 154 | 77 | 50·0 | 0·75 | 0·54 | 1·05 | 0·09 | 88 | 54 | 61·4 | 2·25 | 1·45 | 3·55 | **<0·001** |
| **Chronic respiratory disease^a^** | |  |  |  |  |  |  |  |  |  |  |  |  |  |  |
| No | | 1485 | 833 | 56·1 | 1 |  |  |  | 998 | 406 | 40·7 | 1 |  |  |  |
| Yes | | 297 | 189 | 63·6 | 1·37 | 1·060 | 1·78 | 0·02 | 185 | 116 | 62·7 | 2·45 | 1·78 | 3·40 | **<0·001** |
| **Renal disease^a^** | |  |  |  |  |  |  |  |  |  |  |  |  |  |  |
| No | | 1673 | 959 | 57·3 | 1 |  |  |  | 1121 | 483 | 43·1 | 1 |  |  |  |
| Yes | | 90 | 56 | 62·2 | 1·22 | 0·79 | 1·91 | 0·36 | 48 | 35 | 72·9 | 3·53 | 1·89 | 7·01 | **<0·001** |
| **Liver disease^a^** | |  |  |  |  |  |  |  |  |  |  |  |  |  |  |
| No | | 1721 | 986 | 57·3 | 1 |  |  |  | 1146 | 502 | 43·8 | 1 |  |  |  |
| Yes | | 52 | 33 | 63·5 | 1·29 | 0·73 | 2·34 | 0·38 | 29 | 19 | 65·5 | 2·42 | 1·13 | 5·50 | 0·02 |
| **HIV** | |  |  |  |  |  |  |  |  |  |  |  |  |  |  |
| No | | 1015 | 615 | 60·6 | 1 |  |  |  | 629 | 316 | 50·2 | 1 |  |  |  |
| Yes | | 38 | 14 | 36·8 | 0·38 | 0·19 | 0·74 | 0·002 | 27 | 10 | 37·0 | 0·59 | 0·25 | 1·29 | 0·19 |
| **Active cancer^a^** | |  |  |  |  |  |  |  |  |  |  |  |  |  |  |
| No | | 1675 | 951 | 56·8 | 1 |  |  |  | 1122 | 478 | 42·6 | 1 |  |  |  |
| Yes | | 121 | 79 | 65·3 | 1·43 | 0·98 | 2·122 | 0·067 | 72 | 49 | 68·1 | 2·858 | 1·734 | 4·845 | **<0·001** |
| **Transplant recipients^a^** | |  |  |  |  |  |  |  |  |  |  |  |  |  |  |
| No | | 1764 | 1011 | 57·3 | 1 |  |  |  | 1172 | 510 | 43·5 | 1 |  |  |  |
| Yes | | 32 | 19 | 59·4 | 1·08 | 0·53 | 2·27 | 0·83 | 22 | 17 | 77·3 | 4·312 | 1·678 | 13·452 | 0·002 |
| **Autoimmune diseases^a^** | |  |  |  |  |  |  |  |  |  |  |  |  |  |  |
| No | | 909 | 575 | 63·3 | 1 |  |  |  | 539 | 291 | 54·0 | 1 |  |  |  |
| Yes | | 95 | 62 | 65·3 | 1·09 | 0·70 | 1·72 | 0·71 | 60 | 45 | 75·0 | 2·54 | 1·41 | 4·82 | 0·002 |
| **Ongoing immuno-suppressive therapy** | |  |  |  |  |  |  |  |  |  |  |  |  |  |  |
| No | | 87 | 37 | 42·5 | 1 |  |  |  | 58 | 23 | 39·7 | 1 |  |  |  |
| Yes | | 50 | 22 | 44·0 | 1·06 | 0·52 | 2·15 | 0·87 | 35 | 22 | 62·9 | 2·54 | 1·08 | 6·20 | 0·03 |
| ***Vaccination*** | | | | | | | | | | | | | | | |
| **≥1 dose before acute infection (breakthrough infections)** | |  |  |  |  |  |  |  |  |  |  |  |  |  |  |
| No | | 1492 | 919 | 61·6 | 1 |  |  |  | 978 | 460 | 47·0 | 1 |  |  |  |
| Yes | | 283 | 104 | 36·7 | 0·36 | 0·28 | 0·47 | **<0·001** | 202 | 65 | 32·2 | 0·54 | 0·39 | 0·74 | **<0·001** |
| **Number of doses before acute infection** | |  |  |  |  |  |  |  |  |  |  |  |  |  |  |
| 0 | | 960 | 549 | 57·2 | 1 |  |  |  | 700 | 277 | 39·6 | 1 |  |  |  |
| 1 | | 66 | 19 | 28·8 | 0·30 | 0·17 | 0·52 | **<0·001** | 46 | 16 | 34·8 | 0·82 | 0·43 | 1·51 | 0·53 |
| ≥2 | | 48 | 9 | 18·8 | 0·18 | 0·08 | 0·35 | **<0·001** | 14 | 5 | 35·7 | 0·86 | 0·26 | 2·56 | 0·79 |
| **≥1 dose after infection** | |  |  |  |  |  |  |  |  |  |  |  |  |  |  |
| No | | 384 | 241 | 62·8 | 1 |  |  |  | 239 | 132 | 55·2 | 1 |  |  |  |
| Yes | | 1081 | 581 | 53·7 | 0·69 | 0·54 | 0·88 | 0·002 | 764 | 300 | 39·3 | 0·53 | 0·39 | 0·70 | **<0·001** |
| **Number of doses after infection** | |  |  |  |  |  |  |  |  |  |  |  |  |  |  |
| 0 | | 7 | 2 | 28·6 | 1 |  |  |  | 6 | 2 | 33·3 | 1 |  |  |  |
| 1 | | 481 | 265 | 55·1 | 2·932 | 0·597 | 22·95 | 0·19 | 337 | 141 | 41·8 | 1·39 | 0·25 | 11·35 | 0·72 |
| ≥2 | | 571 | 301 | 52·7 | 2·666 | 0·544 | 20·87 | 0·23 | 417 | 155 | 37·2 | 1·14 | 0·21 | 9·324 | 0·88 |
| ***Acute infection features*** | | | | | | | | | | | | | | | |
| **Variants of concern^b^** | |  |  |  |  |  |  |  |  |  |  |  |  |  |  |
| Alpha (B·1·1·7) | | 125 | 53 | 42·4 | 1 |  |  |  | 91 | 24 | 26·4 | 1 |  |  |  |
| Other variants^c^ | | 105 | 31 | 29·5 | 0·57 | 0·33 | 0·99 | 0·045 | 54 | 17 | 31·5 | 1·28 | 0·60 | 2·69 | 0·51 |
| **First wave^d^** | |  |  |  |  |  |  |  |  |  |  |  |  |  |  |
| No | | 944 | 465 | 50·7 | 1 |  |  |  | 649 | 229 | 35·3 | 1 |  |  |  |
| Yes | | 852 | 301 | 64·7 | 1·78 | 1·47 | 2·15 | **<0·001** | 545 | 298 | 54·7 | 2·21 | 1·75 | 2·79 | **<0·001** |
| **Hospitalization** | |  |  |  |  |  |  |  |  |  |  |  |  |  |  |
| No admission | | 529 | 264 | 49·9 | 1 |  |  |  | 373 | 92 | 24·7 | 1 |  |  |  |
| Admission to non-intensive ward | | 848 | 496 | 58·5 | 1·41 | 1·14 | 1·76 | 0·002 | 580 | 302 | 52·1 | 3·31 | 2·49 | 4·43 | **<0·001** |
| Admission to ICU | | 419 | 270 | 64·4 | 1·82 | 1·40 | 2·37 | <0·001 | 241 | 133 | 55·2 | 3·75 | 2·66 | 5·32 | **<0·001** |
| ***Acute infection treatment*** | | | | | | | | | | | | | | | |
| **Steroid therapy^a^** | |  |  |  |  |  |  |  |  |  |  |  |  |  |  |
| No | | 868 | 500 | 57·6 | 1 |  |  |  | 560 | 242 | 43·2 | 1 |  |  |  |
| Yes | | 661 | 387 | 58·5 | 1·04 | 0·85 | 1·28 | 0·71 | 439 | 209 | 47·6 | 1·19 | 0·93 | 1·54 | 0·17 |
| **Anticoagulant therapy^a^** | |  |  |  |  |  |  |  |  |  |  |  |  |  |  |
| No | | 522 | 270 | 51·7 | 1 |  |  |  | 347 | 119 | 34·3 | 1 |  |  |  |
| Yes | | 608 | 338 | 55·6 | 1·17 | 0·92 | 1·48 | 0·19 | 397 | 194 | 48·9 | 1·83 | 1·36 | 2·46 | **<0·001** |
| **Immunomodulator therapy^a^** | |  |  |  |  |  |  |  |  |  |  |  |  |  |  |
| No | | 1399 | 807 | 57·7 | 1 |  |  |  | 906 | 404 | 44·6 | 1 |  |  |  |
| Yes | | 85 | 55 | 64·7 | 1·34 | 0·85 | 2·15 | 0·20 | 64 | 32 | 50·0 | 1·24 | 0·75 | 2·07 | 0·40 |
| **Monoclonal antibodies therapy^a^** | |  |  |  |  |  |  |  |  |  |  |  |  |  |  |
| No | | 1524 | 945 | 62·0 | 1 |  |  |  | 1027 | 484 | 47·1 | 1 |  |  |  |
| Yes | | 123 | 24 | 19·5 | 0·15 | 0·09 | 0·23 | **<0·001** | 49 | 14 | 28·6 | 0·45 | 0·23 | 0·84 | 0·01 |
| ***Acute infection clinical presentation*** | | | | | | | | | | | | | | | |
| **Number of symptoms** | |  |  |  |  |  |  |  |  |  |  |  |  |  |  |
| 0 | | 47 | 27 | 57·4 |  |  |  |  | 31 | 16 | 51·6 | 1 |  |  |  |
| 1 | | 77 | 38 | 49·4 | 0·73 | 0·35 | 1·51 | 0·39 | 43 | 26 | 60·5 | 1·43 | 0·56 | 3·69 | 0·46 |
| 2 | | 150 | 76 | 50·7 | 0·76 | 0·39 | 1·48 | 0·42 | 92 | 55 | 59·8 | 1·39 | 0·61 | 3·19 | 0·44 |
| 3-5 | | 750 | 406 | 54·1 | 0·88 | 0·48 | 1·59 | 0·66 | 487 | 200 | 41·1 | 0·65 | 0·31 | 1·37 | 0·26 |
| 6-8 | | 488 | 303 | 62·1 | 1·22 | 0·65 | 2·23 | 0·53 | 329 | 142 | 43·2 | 0·71 | 0·34 | 1·51 | 0·37 |
| >9 | | 197 | 128 | 65·0 | 1·37 | 0·71 | 2·63 | 0·34 | 149 | 64 | 43·0 | 0·71 | 0·32 | 1·55 | 0·39 |
| **General symptoms^a^** | |  |  |  |  |  |  |  |  |  |  |  |  |  |  |
| No | | 45 | 23 | 51·1 | 1 |  |  |  | 27 | 14 | 51·9 | 1 |  |  |  |
| Yes | | 1673 | 957 | 57·2 | 1·28 | 0·70 | 2·33 | 0·42 | 1116 | 483 | 43·3 | 0·71 | 0·32 | 1·54 | 0·38 |
| **Respiratory symptoms^a^** | |  |  |  |  |  |  |  |  |  |  |  |  |  |  |
| No | | 148 | 74 | 50·0 | 1 |  |  |  | 103 | 29 | 28·2 | 1 |  |  |  |
| Yes | | 1498 | 870 | 58·1 | 1·39 | 0·99 | 1·95 | 0·06 | 1001 | 449 | 44·9 | 2·07 | 1·34 | 3·28 | **0·001** |
| **Gastrointestinal symptoms^a^** | |  |  |  |  |  |  |  |  |  |  |  |  |  |  |
| No | | 1017 | 551 | 54·2 | 1 |  |  |  | 684 | 296 | 43·3 | 1 |  |  |  |
| Yes | | 662 | 407 | 61·5 | 1·35 | 1·11 | 1·65 | 0·003 | 436 | 196 | 45·0 | 1·07 | 0·84 | 1·36 | 0·58 |
| **Neurological symptoms^a^** | |  |  |  |  |  |  |  |  |  |  |  |  |  |  |
| No | | 285 | 103 | 36·1 | 1 |  |  |  | 211 | 74 | 35·1 | 1 |  |  |  |
| Yes | | 779 | 477 | 61·2 | 2·79 | 2·11 | 3·70 | **<0·001** | 543 | 217 | 40·0 | 1·231 | 0·87 | 1·720 | 0·22 |
| ***Acute infection complications*** | | | | | | | | | | | | | | | |
| **Pulmonary complications^a^** | |  |  |  |  |  |  |  |  |  |  |  |  |  |  |
| No | | 1606 | 939 | 58·5 | 1 |  |  |  | 1051 | 480 | 45·7 | 1 |  |  |  |
| Yes | | 47 | 30 | 63·8 | 1·25 | 0·69 | 2·34 | 0·47 | 28 | 17 | 60·7 | 1·83 | 0·85 | 4·09 | 0·12 |
| **Cardiac complications^a^** | |  |  |  |  |  |  |  |  |  |  |  |  |  |  |
| No | | 1558 | 908 | 58·3 | 1 |  |  |  | 1024 | 466 | 45·5 | 1 |  |  |  |
| Yes | | 97 | 62 | 63·9 | 1·27 | 0·83 | 1·96 | 0·28 | 57 | 33 | 57·9 | 1·64 | 0·96 | 2·85 | 0·07 |
| **Embolic complications^a^** | |  |  |  |  |  |  |  |  |  |  |  |  |  |  |
| No | | 1562 | 909 | 58·2 | 1 |  |  |  | 1027 | 470 | 45·8 | 1 |  |  |  |
| Yes | | 90 | 60 | 66·7 | 1·43 | 0·92 | 2·28 | 0·11 | 52 | 28 | 53·8 | 1·38 | 0·79 | 2·44 | 0·26 |
| **Neurological complications^a^** | |  |  |  |  |  |  |  |  |  |  |  |  |  |  |
| No | | 1644 | 962 | 58·5 | 1 |  |  |  | 1074 | 496 | 46·2 | 1 |  |  |  |
| Yes | | 9 | 7 | 77·8 | 2·354 | 0·55 | 17·47 | 0·27 | 6 | 3 | 50·0 | 1·17 | 0·20 | 6·81 | 0·86 |
| **Renal complications^a^** | |  |  |  |  |  |  |  |  |  |  |  |  |  |  |
| No | | 1552 | 903 | 58·2 | 1 |  |  |  | 1018 | 456 | 44·8 | 1 |  |  |  |
| Yes | | 100 | 64 | 64·0 | 1·28 | 0·84 | 1·96 | 0·25 | 61 | 43 | 70·5 | 2·93 | 1·69 | 5·28 | **<0·001** |
| **Gastrointestinal complications^a^** | |  |  |  |  |  |  |  |  |  |  |  |  |  |  |
| No | | 1528 | 898 | 58·8 | 1 |  |  |  | 1000 | 460 | 46·0 | 1 |  |  |  |
| Yes | | 127 | 71 | 55·9 | 0·89 | 0·62 | 1·29 | 0·53 | 81 | 39 | 48·1 | 1·09 | 0·69 | 1·72 | 0·71 |

^a^See table S1 for definitions; ^b^analysis performed on samples available at baseline; ^c^20A, 20B, 20E (EU1); ^d^SARS-CoV-2 test performed before 1^st^ September 2020; ICU – intensive care units

## Table S6. Comparison of demographic and epidemiological characteristics between females and male patients with SARS-CoV-2 included in the cohort and followed-up to 12 months

| **Variable** | **Total** | **Female** | **%** | **Crude OR** | **95% CI** | | ***p-*value** |
| --- | --- | --- | --- | --- | --- | --- | --- |
|  |  |  |  |  | **lower** | **upper** |  |
| ***Demographic and clinical characteristics*** | | | | | | | |
| **Age groups (years old)** |  |  |  |  |  |  |  |
| 15-30 | 98 | 59 | 60·2 | 1 |  |  |  |
| 31-40 | 150 | 75 | 50·0 | 0·66 | 0·39 | 1·11 | 0·12 |
| 41-60 | 773 | 356 | 46·1 | 0·57 | 0·37 | 0·87 | **0·01** |
| 61-80 | 689 | 256 | 37·2 | 0·39 | 0·25 | 0·60 | **<0·001** |
| >80 | 83 | 31 | 37·3 | 0·40 | 0·22 | 0·72 | **0·002** |
| **Smoking status** |  |  |  |  |  |  |  |
| Non-smoker | 1039 | 514 | 49·5 | 1 |  |  |  |
| Former smoker | 434 | 136 | 31·3 | 0·47 | 0·37 | 0·59 | **<0·001** |
| Smoker | 112 | 51 | 45·5 | 0·85 | 0·58 | 1·26 | 0·43 |
| **Body Mass Index** |  |  |  |  |  |  |  |
| <30 | 263 | 91 | 34·6 | 1 |  |  |  |
| ≥30 | 138 | 60 | 43·5 | 1·45 | 0·95 | 2·22 | 0·08 |
| **Cardiovascular diseases^a^** |  |  |  |  |  |  |  |
| No | 1011 | 487 | 48·2 | 1 |  |  |  |
| Yes | 710 | 270 | 38·0 | 0·66 | 0·54 | 0·80 | **<0·001** |
| **Cardiovascular disease classification** |  |  |  |  |  |  |  |
| None | 1011 | 487 | 48·2 | 1 |  |  |  |
| Congestive heart failure | 9 | 3 | 33·3 | 0·55 | 0·11 | 2·17 | 0·40 |
| Coronary heart disease | 175 | 46 | 26·3 | 0·39 | 0·27 | 0·55 | **<0·001** |
| Hypertension | 468 | 200 | 42·7 | 0·80 | 0·64 | 1·00 | 0·05 |
| Other | 58 | 21 | 36·2 | 0·61 | 0·35 | 1·05 | 0·08 |
| **Diabetes** |  |  |  |  |  |  |  |
| No | 1544 | 692 | 44·8 | 1 |  |  |  |
| Yes | 154 | 48 | 31·2 | 0·56 | 0·39 | 0·79 | **<0·001** |
| **Chronic respiratory disease^a^** |  |  |  |  |  |  |  |
| No | 1484 | 650 | 43·8 | 1 |  |  |  |
| Yes | 297 | 122 | 41·1 | 0·90 | 0·69 | 1·15 | 0·39 |
| **Renal disease^a^** |  |  |  |  |  |  |  |
| No | 1672 | 742 | 44·4 | 1 |  |  |  |
| Yes | 90 | 17 | 18·9 | 0·29 | 0·17 | 0·49 | **<0·001** |
| **Liver disease^a^** |  |  |  |  |  |  |  |
| No | 1720 | 743 | 43·2 | 1 |  |  |  |
| Yes | 52 | 22 | 42·3 | 0·97 | 0·55 | 1·69 | 0·90 |
| **HIV infection** |  |  |  |  |  |  |  |
| No | 1014 | 413 | 40·7 | 1 |  |  |  |
| Yes | 38 | 5 | 13·2 | 0·23 | 0·08 | 0·54 | **<0·001** |
| **Active cancer^a^** |  |  |  |  |  |  |  |
| No | 1674 | 732 | 43·7 | 1 |  |  |  |
| Yes | 121 | 47 | 38·8 | 0·82 | 0·56 | 1·19 | 0·30 |
| **Transplant recipients^a^** |  |  |  |  |  |  |  |
| No | 1763 | 769 | 43·6 | 1 |  |  |  |
| Yes | 32 | 10 | 31·3 | 0·59 | 0·27 | 1·23 | 0·17 |
| **Autoimmune diseases^a^** |  |  |  |  |  |  |  |
| No | 909 | 322 | 35·4 | 1 |  |  |  |
| Yes | 95 | 47 | 49·5 | 1·78 | 1·16 | 2·73 | **0·01** |
| **Ongoing immuno-suppressive therapy** |  |  |  |  |  |  |  |
| No | 87 | 36 | 41·4 | 1 |  |  |  |
| Yes | 50 | 23 | 46·0 | 1·21 | 0·59 | 2·44 | 0·60 |
| ***Vaccination*** | | | | | | | |
| **≥1 dose before acute infection (breakthrough infections)** |  |  |  |  |  |  |  |
| No | 1492 | 626 | 42·0 | 1 |  |  |  |
| Yes | 282 | 141 | 50·0 | 1·38 | 1·07 | 1·77 | **0·01** |
| **Number of doses before acute infection (breakthrough infections)** |  |  |  |  |  |  |  |
| 0 | 959 | 438 | 45·7 | 1 |  |  |  |
| 1 | 66 | 32 | 48·5 | 1·12 | 0·68 | 1·85 | 0·66 |
| ≥2 | 48 | 27 | 56·3 | 1·53 | 0·85 | 2·77 | 0·16 |
| **≥1 dose after infection** |  |  |  |  |  |  |  |
| No | 384 | 157 | 40·9 | 1 |  |  |  |
| Yes | 1080 | 499 | 46·2 | 1·24 | 0·98 | 1·57 | 0·07 |
| **Number of doses after acute infection** |  |  |  |  |  |  |  |
| 0 | 7 | 3 | 42·9 | 1 |  |  |  |
| 1 | 481 | 210 | 43·7 | 1·02 | 0·21 | 5·59 | 0·98 |
| ≥2 | 570 | 278 | 48·8 | 1·25 | 0·26 | 6·86 | 0·78 |
| ***Acute infection features*** | | | | | | | |
| **Variants of concern^b^** |  |  |  |  |  |  |  |
| Alpha (B·1·1·7) | 125 | 67 | 53·6 | 1 |  |  |  |
| Other variants^c^ | 104 | 47 | 45·2 | 0·72 | 0·42 | 1·21 | 0·21 |
| **First wave^d^** |  |  |  |  |  |  |  |
| No | 943 | 469 | 49·7 | 1 |  |  |  |
| Yes | 852 | 310 | 36·4 | 0·58 | 0·48 | 0·79 | **<0·001** |
| **Hospitalization** |  |  |  |  |  |  |  |
| No admission | 528 | 316 | 59·8 | 1 |  |  |  |
| Admission to non-intensive ward | 848 | 348 | 41·0 | 0·47 | 0·37 | 0·58 | **<0·001** |
| Admission to ICU | 419 | 115 | 27·4 | 0·25 | 0·19 | 0·34 | **<0·001** |
| ***Acute infection treatment*** | | | | | | | |
| **Steroid therapy^a^** |  |  |  |  |  |  |  |
| No | 868 | 404 | 46·5 | 1 |  |  |  |
| Yes | 661 | 255 | 38·6 | 0·72 | 0·59 | 0·89 | **0·002** |
| **Anticoagulant therapy^a^** |  |  |  |  |  |  |  |
| No | 522 | 281 | 53·8 | 1 |  |  |  |
| Yes | 608 | 232 | 38·2 | 0·53 | 0·42 | 0·67 | **<0·001** |
| **Immunomodulator therapy^a^** |  |  |  |  |  |  |  |
| No | 1399 | 620 | 44·3 | 1 |  |  |  |
| Yes | 85 | 24 | 28·2 | 0·50 | 0·30 | 0·80 | **0·003** |
| **Monoclonal antibodies therapy^a^** |  |  |  |  |  |  |  |
| No | 1524 | 641 | 42·1 | 1 |  |  |  |
| Yes | 123 | 54 | 43·9 | 1·08 | 0·74 | 1·56 | 0·69 |
| ***Acute infection clinical presentation*** | | | | | | | |
| **Number of symptoms** |  |  |  |  |  |  |  |
| 0 | 47 | 16 | 34·0 | 1 |  |  |  |
| 1 | 77 | 30 | 39·0 | 1·23 | 0·58 | 2·68 | 0·60 |
| 2 | 150 | 51 | 34·0 | 1.0 | 0·50 | 2·03 | 0·99 |
| 3-5 | 750 | 265 | 35·3 | 1·05 | 0·57 | 2·02 | 0·87 |
| 6-8 | 488 | 238 | 48·8 | 1·84 | 0·99 | 3·53 | 0·06 |
| >9 | 196 | 130 | 66·3 | 3·78 | 1·95 | 7·59 | **<0·001** |
| **General symptoms^a^** |  |  |  |  |  |  |  |
| No | 45 | 13 | 28·9 | 1 |  |  |  |
| Yes | 1672 | 734 | 43·9 | 1·91 | 1·02 | 3·82 | **0·04** |
| **Respiratory symptoms^a^** |  |  |  |  |  |  |  |
| No | 148 | 73 | 49·3 | 1 |  |  |  |
| Yes | 1497 | 648 | 43·3 | 0·78 | 0·56 | 1·10 | 0·16 |
| **Gastrointestinal symptoms^a^** |  |  |  |  |  |  |  |
| No | 1017 | 383 | 37·7 | 1 |  |  |  |
| Yes | 661 | 354 | 53·6 | 1·91 | 1·57 | 2·33 | **<0·001** |
| **Neurological symptoms^a^** |  |  |  |  |  |  |  |
| No | 285 | 112 | 39·3 | 1 |  |  |  |
| Yes | 778 | 402 | 51·7 | 1·65 | 1·25 | 2·18 | **<0·001** |
| ***Acute infection complications*** | | | | | | | |
| **Pulmonary complications^a^** |  |  |  |  |  |  |  |
| No | 1606 | 687 | 42·8 | 1 |  |  |  |
| Yes | 47 | 11 | 23·4 | 0·41 | 0·20 | 0·79 | **0·01** |
| **Cardiac complications^a^** |  |  |  |  |  |  |  |
| No | 1558 | 672 | 43·1 | 1 |  |  |  |
| Yes | 97 | 27 | 27·8 | 0·51 | 0·32 | 0·80 | **0·003** |
| **Embolic complications^a^** |  |  |  |  |  |  |  |
| No | 1562 | 668 | 42·8 | 1 |  |  |  |
| Yes | 90 | 30 | 33·3 | 0·67 | 0·422 | 1·04 | 0·08 |
| **Neurological complications^a^** |  |  |  |  |  |  |  |
| No | 1644 | 696 | 42·3 | 1 |  |  |  |
| Yes | 9 | 2 | 22·2 | 0·41 | 0·06 | 1·76 | 0·25 |
| **Renal complications^a^** |  |  |  |  |  |  |  |
| No | 1552 | 674 | 43·4 | 1 |  |  |  |
| Yes | 100 | 24 | 24·0 | 0·41 | 0·25 | 0·65 | **<0·001** |
| **Gastrointestinal complications^a^** |  |  |  |  |  |  |  |
| No | 1528 | 663 | 43·4 | 1 |  |  |  |
| Yes | 127 | 35 | 27·6 | 0·50 | 0·33 | 0·74 | **<0·001** |

^a^See table S1 for definitions; ^b^analysis performed on samples available at baseline; ^c^20A, 20B, 20E (EU1); ^d^SARS-CoV-2 test performed before 1^st^ September 2020; ICU – intensive care unit.

## Table S8. Comparison of demographic and epidemiological characteristics between hospitalized and non-hospitalized patients with SARS-CoV-2 included in the cohort and followed-up to 12 months

| **Variable** | **Total** | **Hospitalized** | **%** | **Crude OR** | **95% CI** | | **p-value** |
| --- | --- | --- | --- | --- | --- | --- | --- |
|  |  |  |  |  | **lower** | **upper** |  |
| *Demographic and clinical characteristics* | | | | | | | |
| **Sex** |  |  |  |  |  |  |  |
| Male | 1016 | 804 | 79·1 | 1 |  |  |  |
| Female | 779 | 463 | 59·4 | 0·39 | 0·31 | 0·48 | <0·001 |
| **Age groups (years old)** |  |  |  |  |  |  |  |
| 15-30 | 98 | 23 | 23·5 | 1 |  |  |  |
| 31-40 | 150 | 81 | 54·0 | 3·79 | 2·17 | 6·81 | <0·001 |
| 41-60 | 773 | 512 | 66·2 | 6·33 | 3·93 | 10·56 | <0·001 |
| 61-80 | 689 | 572 | 83·0 | 15·79 | 9·64 | 26·80 | <0·001 |
| >80 | 83 | 79 | 95·2 | 60·38 | 22·11 | 219·03 | <0·001 |
| **Cardiovascular diseases^a^** |  |  |  |  |  |  |  |
| No | 1012 | 620 | 61·3 | 1 |  |  |  |
| Yes | 710 | 583 | 82·1 | 2·90 | 2·31 | 3·66 | <0·001 |
| **Diabetes** |  |  |  |  |  |  |  |
| No | 1545 | 1043 | 67·5 | 1 |  |  |  |
| Yes | 154 | 134 | 87·0 | 3·20 | 2·02 | 5·34 | <0·001 |
| **Chronic respiratory diseases^a^** |  |  |  |  |  |  |  |
| No | 1485 | 1014 | 68·3 | 1 |  |  |  |
| Yes | 297 | 245 | 82·5 | 2·18 | 1·60 | 3·03 | <0·001 |
| **Renal diseases^a^** |  |  |  |  |  |  |  |
| No | 1673 | 1172 | 70·1 | 1 |  |  |  |
| Yes | 90 | 86 | 95·6 | 8·85 | 3·67 | 29·61 | <0·001 |
| Vaccination | | | | | | | |
| **≥1 dose before acute infection (breakthrough infections)** |  |  |  |  |  |  |  |
| No | 1492 | 1194 | 80·0 | 1 |  |  |  |
| Yes | 283 | 70 | 24·7 | 0·08 | 0·06 | 0·11 | <0·001 |
| **Number of doses before acute infection (breakthrough infections)** |  |  |  |  |  |  |  |
| 0 | 960 | 582 | 60·6 | 1 |  |  |  |
| 1 | 66 | 26 | 39·4 | 0·42 | 0·25 | 0·70 | <0·001 |
| ≥2 | 48 | 10 | 20·8 | 0·17 | 0·08 | 0·34 | <0·001 |
| *Acute infection features* | | | | | | | |
| **First wave^d^** |  |  |  |  |  |  |  |
| No | 944 | 436 | 46·2 | 1 |  |  |  |
| Yes | 852 | 831 | 97·5 | 45·7 | 29·8 | 74.00 | <0·001 |
| **Monoclonal antibodies therapy^a^** |  |  |  |  |  |  |  |
| No | 1524 | 1232 | 80·8 | 1 |  |  |  |
| Yes | 123 | 21 | 17·1 | 0·05 | 0·03 | 0·08 | <0·001 |
| *Acute infection clinical presentation* | | | | | | | |
| **Number of symptoms** |  |  |  |  |  |  |  |
| 0 | 47 | 13 | 72·3 | 1 |  |  |  |
| 1 | 77 | 20 | 74·0 | 1·09 | 0·47 | 2·47 | 0·84 |
| 2 | 150 | 30 | 80·0 | 1.53 | 0·70 | 3·23 | 0·28 |
| 3-5 | 750 | 141 | 81·2 | 1·66 | 0·82 | 3·17 | 0·15 |
| 6-8 | 488 | 162 | 66·8 | 0·78 | 0·38 | 1·48 | 0·45 |
| >9 | 197 | 125 | 36·5 | 0·22 | 0·11 | 0·44 | <0·001 |
| **General symptoms^a^** |  |  |  |  |  |  |  |
| No | 45 | 23 | 51·1 | 1 |  |  |  |
| Yes | 1673 | 1177 | 70·4 | 2·27 | 1·24 | 4·14 | 0·00 |
| **Respiratory symptoms^a^** |  |  |  |  |  |  |  |
| No | 148 | 3 | 35·8 | 1 |  |  |  |
| Yes | 1498 | 1095 | 73·1 | 4·86 | 3·42 | 6·97 | <0·001 |
| **Gastrointestinal symptoms^a^** |  |  |  |  |  |  |  |
| No | 1017 | 710 | 69·8 | 1 |  |  |  |
| Yes | 662 | 465 | 70·2 | 1·02 | 0·82 | 1·27 | 0·86 |
| **Neurological symptoms^a^** |  |  |  |  |  |  |  |
| No | 285 | 157 | 55·1 | 1 |  |  |  |
| Yes | 779 | 430 | 55·2 | 1·01 | 0·76 | 1·32 | 0·97 |
| ^a^See table S1 for definitions; ^d^SARS-CoV-2 test performed before 1^st^ September 2020 | | | | | | | |

## Figure S2. Differences in blood test parameters between patients with (red) and without (blue) at least one symptom at 6- and 12-month follow-up


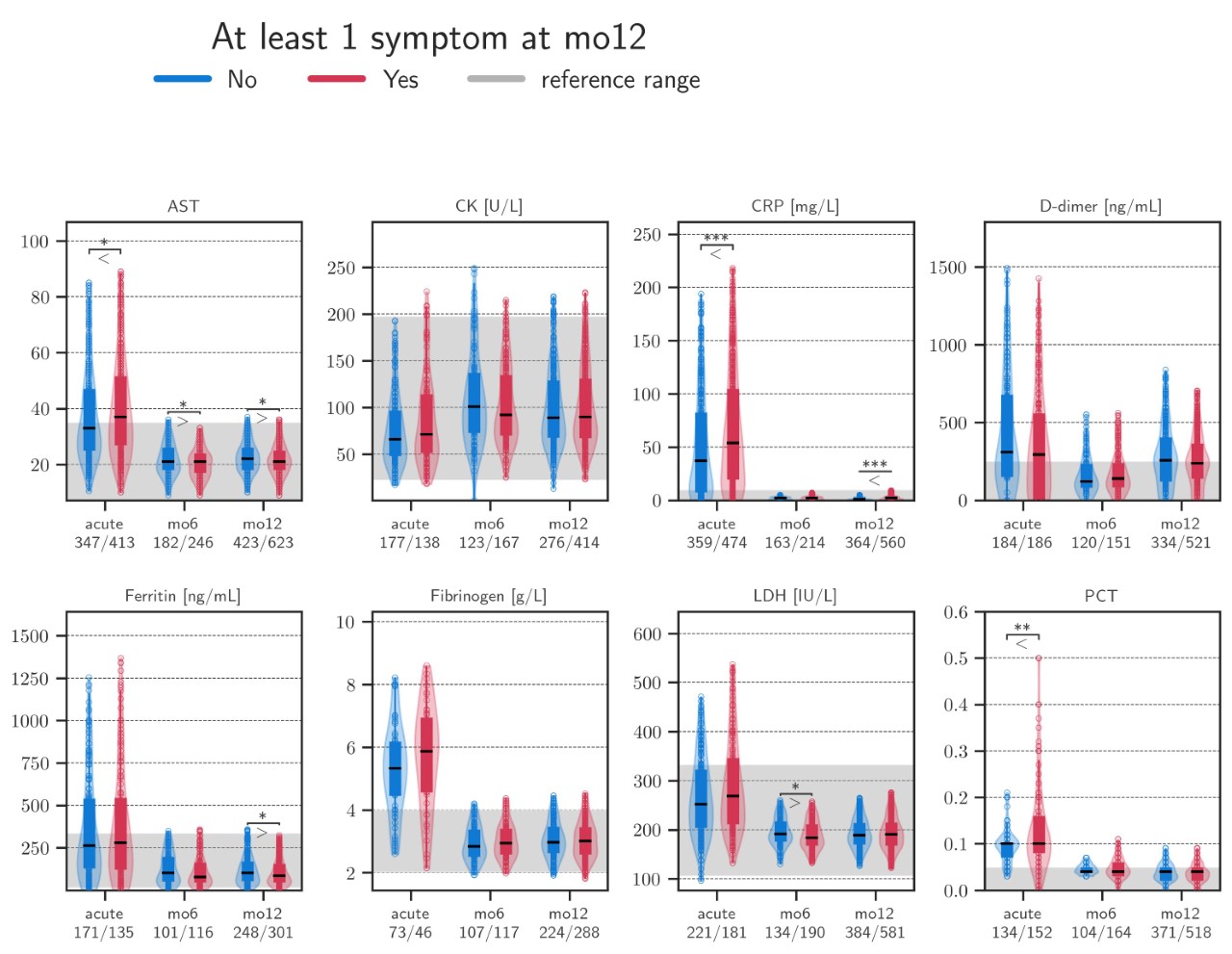


AST: aspartate aminotransferase (U/L); CK: Creatine kinase (U/L); CRP: C-reactive Protein (mg/L); LDH: lactate dehydrogenase (U/L); PCT: procalcitonin (ng/mL); *** ≤ 0·001, ** ≤ 0·010, * ≤ 0·050.

## Figure S3. Density plot of SF-36 PCS and MCS scores in 1193 patients at 12-month follow-up


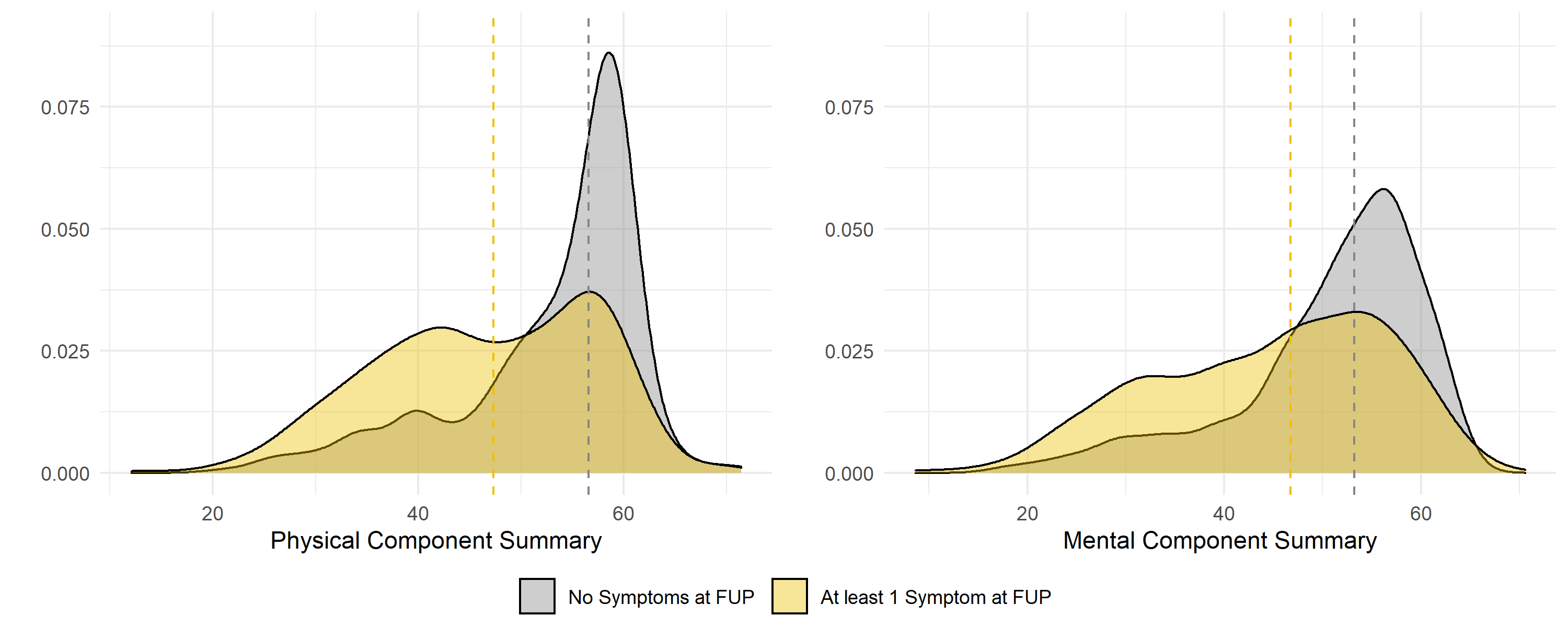


Yellow: patients with PCS; grey: control group. The dashed lines depict group medians.

## Figure S4. Severity of post-COVID-19 syndrome by symptom cluster and quality of life reported by distribution of the physical and mental components of the SF-36 questionnaire at 12-month follow-up

Comparisons: *** ≤ 0·001, ** ≤ 0·010, * ≤ 0·050 (details of methodology used in the main test).


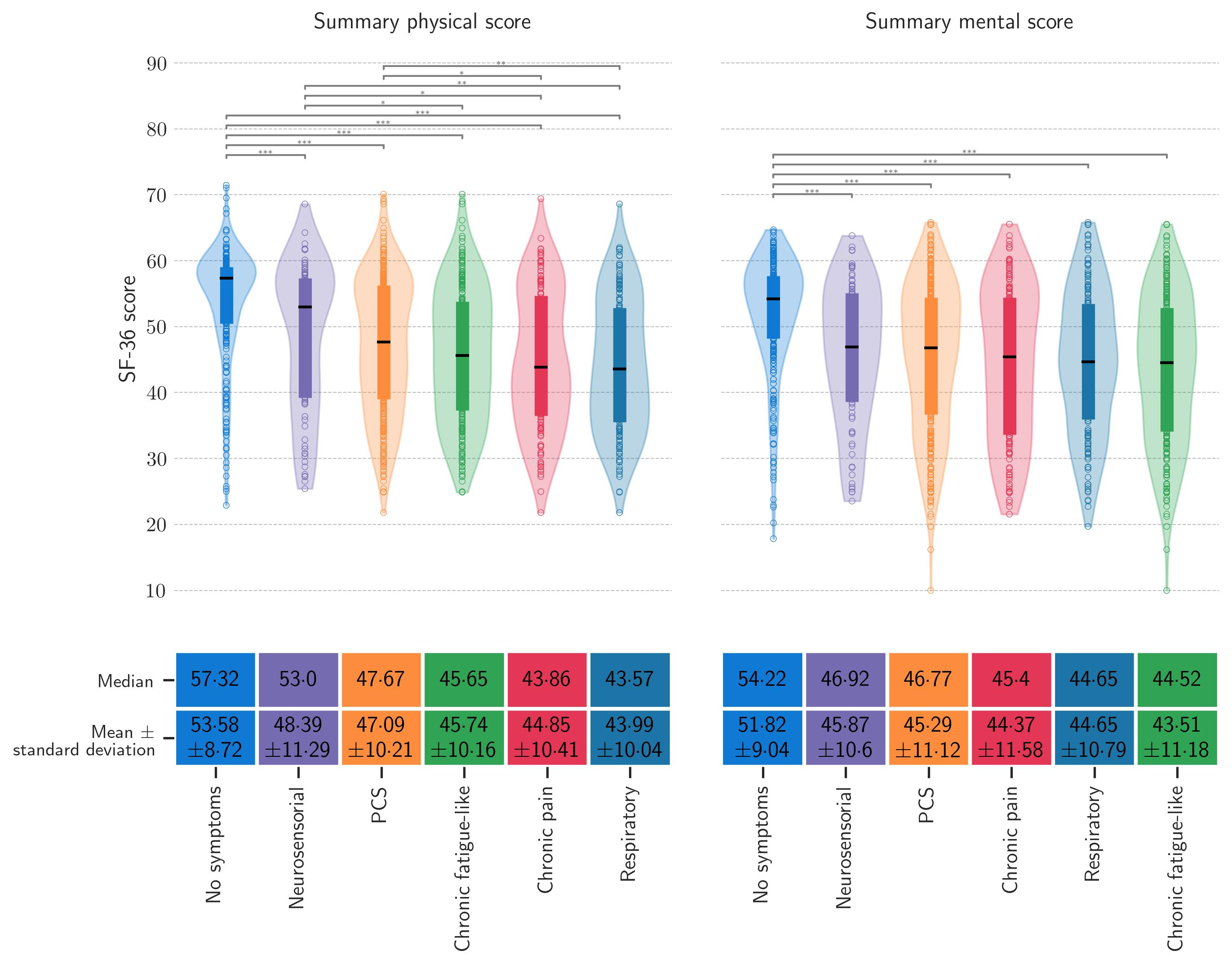


## Figure S5. Results of the univariable analysis of factors associated with the four clinical phenotypes: respiratory, neurosensorial, chronic pain and chronic fatigue-like


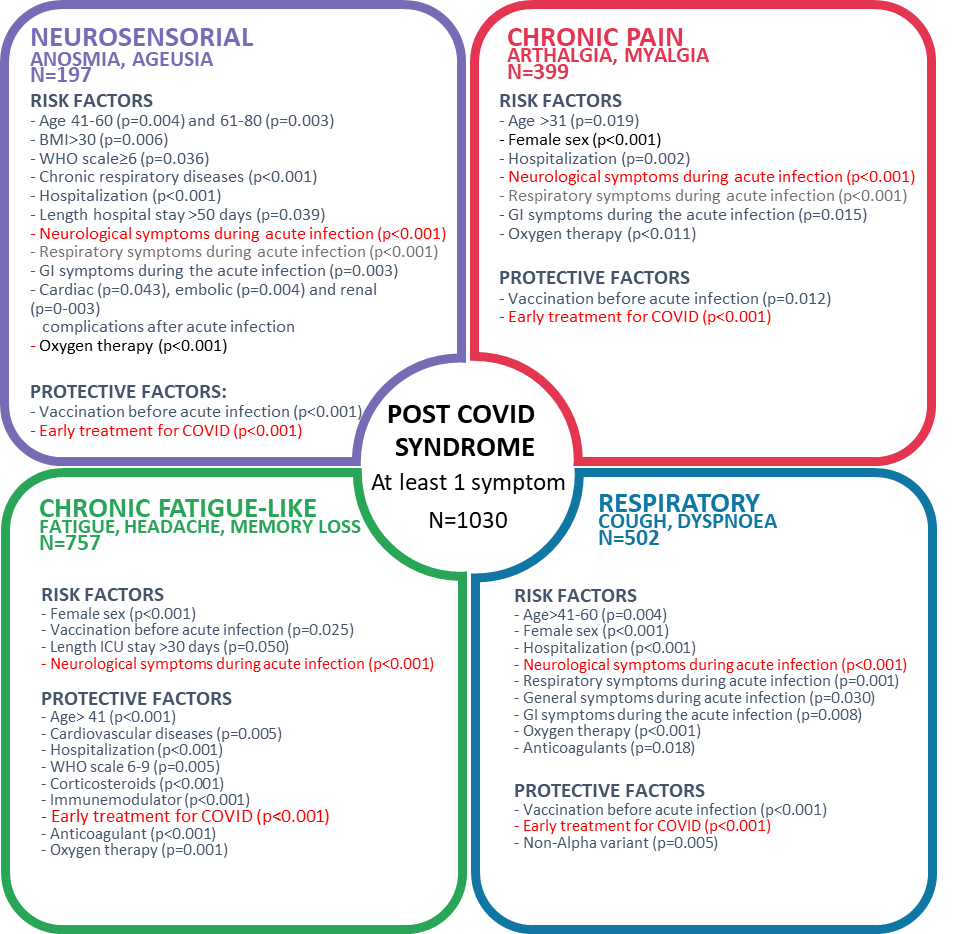


Red: variables significantly associated with occurrence of all the four clusters.

## Figure S6. Differences in anti-S response between patients with and without post-COVID-19 syndrome at 12-month follow-up according to time since last vaccination against SARS-CoV-2


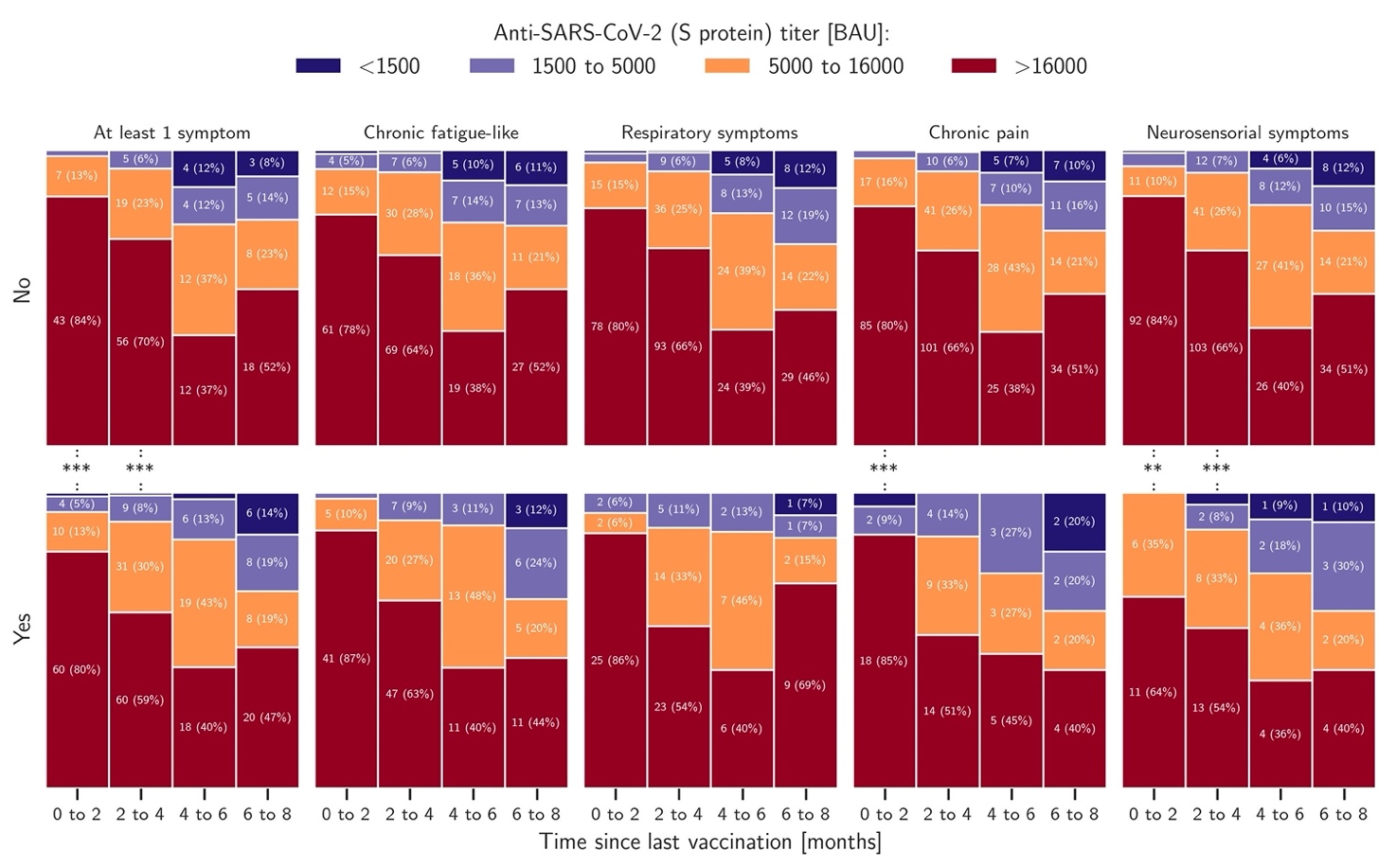


Anti-S titer is provided in BAU. The numbers denote the sample size for each comparison. Comparison: *** ≤ 0·001, ** ≤ 0·010, * ≤ 0·050 (details on statistical methodology in the main manuscript – statistical analysis section)

## Figure S7. Severity of post-COVID-19 syndrome according to clinical phenotype and quality of life reported by distribution of the physical component of the SF-36 questionnaire and subdomains (physical functioning, role limitations due to physical health, pain, and general health) at 12-month follow up


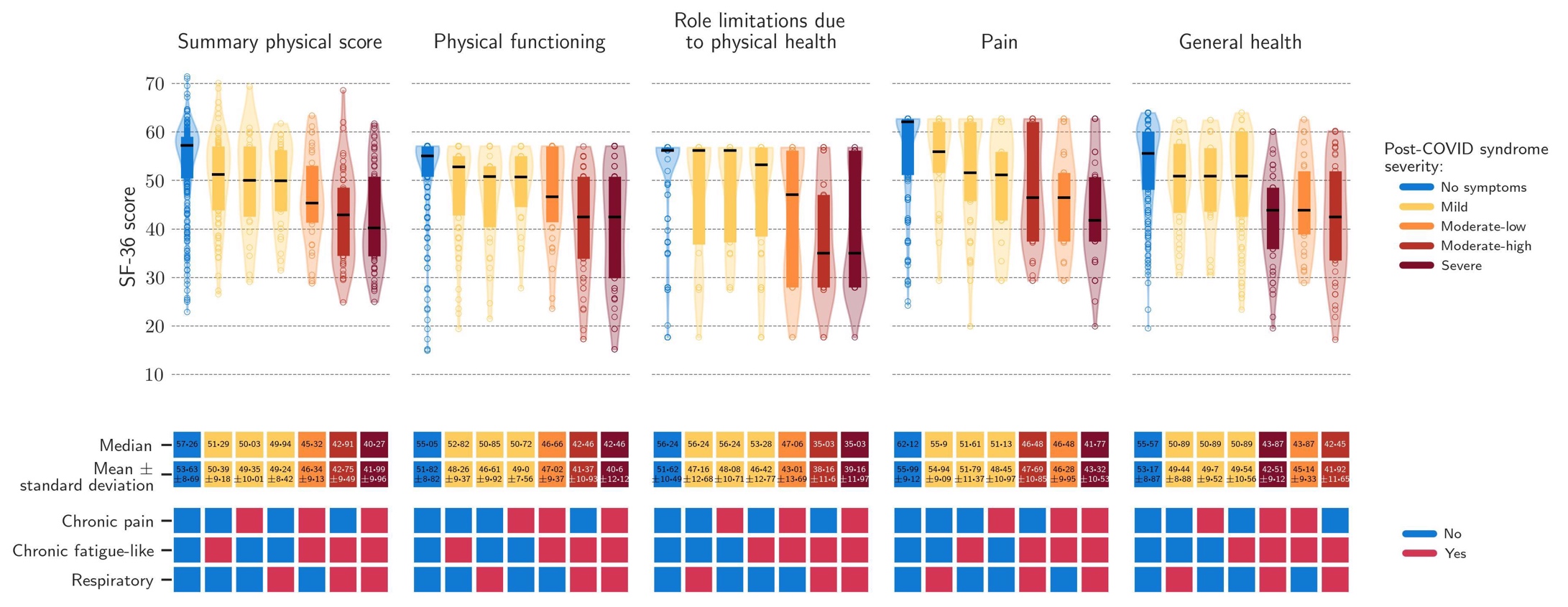


Patients are clustered into only one clinical phenotype or combination of clinical phenotypes.

## SECTION 4: LITERATURE REVIEW

## Table S8. Literature review of systematic reviews and meta-analysis reporting on long COVID symptoms at 12 months after acute infection

Research strategy: "post-acute COVID-19 syndrome" [Supplementary Concept] AND symptom

Time: until October 2022

Language: no restriction

| **Author** | **Journal** | **Age, years** | **Timepoint** | **Severity** | **Symptoms** | **Number of studies** | **Number of patients** | **Pooled prevalence, CI: confidence interval, I^2^ (detection of heterogeneity)** |
| --- | --- | --- | --- | --- | --- | --- | --- | --- |
| Pinzon RT^1^ | *J Infect Public Health* | >18 | < 6 months | Any | Cognitive disorders: brain fog, difficult thinking, poor attention, memory impairment and other cognitive impairment issues | 5 | 3305 | 35.4%; 95% CI 2.1-81.7, I^2^ 99.7% |
|  |  |  |  |  | Fatigue | 9 | 4546 | 52.8%; 95% CI 19.9-84.4, I^2^ 99.8% |
|  |  |  |  |  | Paresthesia | 3 | 1939 | 33.3%; 95% CI 2.7-76.6, I^2^ 99.8% |
|  |  |  |  |  | Sleep disorder | 5 | 3485 | 32.9%; 95% CI 6.5-67.4, I^2^ 99.8% |
|  |  |  |  |  | Musculoskeletal pain | 3 | 3918 | 27.8%; 95% CI 12.7-45.9 |
|  |  |  |  |  | Dizziness | 5 | 1783 | 26.4%; 95% CI 4.6-57.9, I^2^ 99.9% |
|  |  |  |  |  | Headache | 9 | 3886 | 21.3%; 95% CI 3.3-48.9, I^2^ 99.9% |
|  |  |  |  |  | Dysnosmia | 11 | 2024 | 17.7%; 95% CI 10.3-26.7, I^2^ 99.8% |
|  |  |  |  |  | Dysgeusia | 9 | 1783 | 16.5%; 95% CI 8.3-27.0, I^2^ 99.1% |
|  |  |  |  |  | Movement disorder | 2 | 32 | 3.6%; 95% CI 2.5-4.9 |
| Lopez-Leon S^2^ | *Sci Rep.* | 0-18 | > 4 months | Any | Mood (sad, tense, angry, depression, anxiety) | 5 | 730 | 16.5%; 95% CI 7.4–28.2, I^2^ 97.5% |
|  |  |  |  |  | Fatigue | 16 | 3015 | 9.7%; 95% CI 4.5–16.5, I^2^ 99.1% |
|  |  |  |  |  | Sleep disorders (e.g., insomnia, hypersomnia, and poor sleep quality) | 8 | 153 | 8.4%; 95% CI 3.4–15.2, I^2^ 93.5% |
|  |  |  |  |  | Headache | 13 | 1875 | 7.8%; 95% CI 4.0–12.7, I^2^ 98.5% |
|  |  |  |  |  | Respiratory symptoms | 9 | 1387 | 7.6%; 95% CI 2.1–15.8, I^2^ 99.2% |
|  |  |  |  |  | Sputum production or nasal congestion | 2 | 11 | 7.5%; 95% CI 3.8–12.4 |
|  |  |  |  |  | Cognitive symptoms (e.g., less concentration, learning difficulties, confusion, and memory loss) | 11 | 1223 | 6.3%; 95% CI 4.–58.4, I^2^ 91.3% |
|  |  |  |  |  | Loss of appetite | 5 | 747 | 6.1%; 95% CI 3.9–8.6, I^2^ 93.5% |
|  |  |  |  |  | Exercise intolerance | 2 | 8 | 5.7%; 95% CI 0.0–19.4, I^2^ 87.8% |
|  |  |  |  |  | Altered smell (e.g., hyposmia, anosmia, hypersomnia, parosmia, and phantom smell) | 10 | 2048 | 5.6%; 95% CI 3.1–8.7, I^2^ 97.1% |
| Nguyen NN^3^ | *Eur J Clin Microbiol Infect Dis*. | Any | 6-12 months | Outpatients | Fatigue | 5 |  | 25-34% |
|  |  |  |  |  | Smell and/or taste disorders | 5 |  | 3-24% |
|  |  |  |  |  | Cough | 5 |  | 2-13% |
|  |  |  |  |  | Dyspnea | 5 |  | 13-22% |
|  |  |  |  |  | Thoracic pain | 5 |  | 9% |
|  |  |  |  |  | Arthralgia | 5 |  | 15% |
| Alkodaymi MS^4^ | *Clin Microbiol Infect*. | Any | 6-9 months | Any | Sleep disorder | 12 | 24200 | 29%; 95% CI 15–45, I^2^ 99.7% |
|  |  |  |  |  | Depression | 6 | 4377 | 23%; 95% CI 21–26, I^2^ 66.3% |
|  |  |  |  |  | Anxiety | 7 | 240756 | 23%; 95% CI 13–33, I^2^ 99.3% |
|  |  |  |  |  | Difficulty concentrating | 4 | 854 | 22%; 95% CI 8–40, I^2^ 96.9% |
|  |  |  |  |  | Cognitive disorder | 5 | 1987 | 15%; 95% CI 6–27, I^2^ 97.6% |
|  |  |  |  |  | Headache | 13 | 7170 | 14%; 95% CI 7–23, I^2^ 99.0% |
|  |  |  |  |  | Loss of smell | 17 | 6596 | 15%; 95% CI 10–22, I^2^ 97.6% |
|  |  |  |  |  | Loss of taste | 16 | 6505 | 13%; 95% CI 8–18, I^2^ 96.9% |
|  |  |  |  |  | Palpitation | 7 | 4735 | 14%; 95% CI 8–21, I^2^ 96.9% |
|  |  |  |  |  | Effort intolerance | 5 | 850 | 45%; 95% CI 25–67, I^2^ 97.4% |
|  |  |  |  |  | Chest pain | 10 | 4318 | 12%; 95% CI 8–18, I^2^ 95.5% |
|  |  |  |  |  | Cough | 21 | 8737 | 12%; 95% CI 6–20, I^2^ 98.0% |
|  |  |  |  |  | Dyspnoea | 13 | 4384 | 25%; 95% CI 20–30, I^2^ 96.8% |
|  |  |  |  |  | Diarrhoea | 8 | 3318 | 5%; 95% CI 2–11, I^2^ 96.4% |
|  |  |  |  |  | Nausea | 8 | 3419 | 4%; 95% CI 1–8, I^2^ 95.2% |
|  |  |  |  |  | Joint pain | 8 | 5288 | 23%; 95% CI 15–31, I^2^ 97.8% |
|  |  |  |  |  | Myalgia | 9 | 3490 | 19%; 95% CI 7–35, I^2^ 99.0% |
|  |  |  |  |  | Fatigue | 19 | 8191 | 36% 95% CI 27–46, I^2^ 98.8% |
|  |  |  |  |  | Hair loss | 5 | 4276 | 10%; 95% CI 2–22, I^2^ 99.2% |
| Ceban F^5^ | *Brain Behav Immun*. | Any | >12 weeks | Any | Cognitive impairment | 43 | 13232 | 22%; 95% CI 1728, I^2^ 98.0% |
|  |  |  |  |  | Fatigue | 68 | 25268 | 32%; 95% CI 27-37, I^2^ 99.1% |
| Renaud-Charest O^6^ | *J Psychiatr Res*. | Any | >12 weeks | Any | Depressive symptoms | 6 | 742 | 11-28% |
|  |  |  |  |  | Clinically-significant depression and/or severe depressive symptoms | 5 | 565 | 3-12% |
| Michelen M^7^ | *BMJ Glob Health.* | Any | 7 months (mean) | Mostly hospitalized | Weakness | 1 | 186 | 41%; 95% CI 25-59, I^2^ 96.0% |
|  |  |  |  |  | General malaise | 2 | 292 | 33%; 95% CI 15-57, I^2^ 97.3% |
|  |  |  |  |  | Fatigue | 17 | 2207 | 31%; 95% CI 24-39, I^2^ 97.9% |
|  |  |  |  |  | Concentration impairment | 2 | 66 | 26%; 95% CI 21-32 |
|  |  |  |  |  | Breathlessness | 20 | 1297 | 25%; 95% CI 18-34, I^2^ 96.0% |
|  |  |  |  |  | Reduced quality of life | 3 | 340 | 37%; 95% CI 18-60, I^2^ 91.0% |
| Cares-Marambio K^8^ | *Chron Respir Dis*. | >18 | 3 weeks-3 months | Hospitalized | Fatigue | 8 | 1668 | 52%; 95% CI 38-66, I^2^ 97.0% |
|  |  |  |  |  | Dyspnoea | 9 | 1334 | 37%; 95% CI 28-48, I^2^ 93.0% |
|  |  |  |  |  | Chest pain | 5 | 1066 | 16%; 95% CI 10-23, I^2^ 90.0% |
|  |  |  |  |  | Cough | 7 | 1496 | 14%; 95% CI 6-24, I^2^ 96.0% |

## Table S9. Literature review of studies assessing the impact of post-COVID-19 syndrome on the quality of life (QoL)

Research strategy: Long COVID AND quality of life (MeSH) + other references found.

Time: until October 2022

Language: no restriction

| **Author** | **Journal** | **Design** | **QoL test** | **Number of participants** | **Timepoint** | **Major results** |
| --- | --- | --- | --- | --- | --- | --- |
| Tsuzuki S^9^ | *Health Qual Life Outcomes* | Cross sectional self-report questionnaire survey | EQ-VAS  EQ-5D-3L | 526 | 250 days | EQ-VAS participants reporting symptoms: 69.9  EQ-VAS participants reporting no symptoms: 82.8  EQ-5D-3L participants reporting symptoms: 0.85  EQ-5D-3L participants reporting no symptoms 0.96 |
| Malik P^10^ | *J Med Virol.* | Systematic review and meta‐analysis | EQ‐VAS | 4828  12 studies | 35-151 days | Pooled EQ‐VAS: 59% (95%CI: 42%–75%) |
| Taboada M^11^ | *Br J Anaesth*. | Prospective questionnaire 6 months after ICU treatment | EQ-5D-3L  EQ‐VAS  PCFS | 91 | 6 months | EQ-VAS: 87.6-66.36 |
| Huang C^12^ | *Lancet* | Ambi-directional cohort study | EQ-VAS  EQ-5D-3L | 1733 | 1 year | EQ-VAS: 80 |
| Catalan IP^13^ | *J Med Virol.* | Observational cohort study (telephone survey) | SF-36 | 76 | 1 year | SF-36 MCS patients non-receiving steroids: 76  SF-36 MCS patients receiving steroids: 86 |
| Tabacof L^14^ | Am J Phys *Med Rehabil.* | Cross-sectional observational study design  (self-administered web-based survey) | EQ-5D-5L  EQ-VAS | 156 |  | EQ-VAS: 64 (6–99) |
| Poudel AN^15^ | *PLOS One* | Systematic review and meta‐analysis | SF-36  EQ-5D-5L | 12 studies |  | SF-36 for PCS: 60-86  EQ-5D-5L index mean value: 0.61-0.71 |
| McFann K^16^ | *Int J Environ Res Public Health* | Observational, longitudinal cohort | SF-36 | 62 | 0-6 months | Physical Functioning PCS: 57.6 (SD 30.4)  Physical Functioning no PCS: 92.7 (17.0)  Role Limitations due to Physical Health PCS: 41.9 (SD 38.3)  Role Limitations due to Physical Health no PCS: 75.3 (SD 37.1)  Role limitations due to Emotional Problems PCS: 58.2 (SD 40.6)  Role limitations due to Emotional Problems no PCS: 82.2 (SD 28.7)  Energy/Fatigue PCS: 36.4 (SD 21.4)  Energy/Fatigue no PCS: 63.8 (SD 24.5)  Pain PASC: 66.5 (SD 28.9)  Pain no PASC: 83.3 (SD 24.0)  Social Functioning PCS: 69.3 (SD 27.1)  Social Functioning no PCS: 86.6 (SD 19.6)  General Health with PCS: 52.9 (SD 20.8)  General Health no PCS: 79.7 (SD 19.3) |
| Schouborg LB^17^ | *Dan Med J*. | retrospective cohort | Functional status scale  EQ-5D-5L  EQ-VAS | 83 |  | EQ-VAS men: 80.4 (SD 17.8)  EQ-VAS women: 66.9 (SD 23.4) |
| Kim Y^18^ | *Yonsei Med J*. | prospective cohort study | EQ-5D-5L | 170 | 12 months | EQ-VAS PCS: 80 (IQR 70-90)  EQ-VAS no PCS: 90 (IQR 80-95) |
| Munoz-Corona C^19^ | *J Int Med Res*. | Observational, ambispective, longitudinal analytic study | SF-36 | 141 | 90 days | ICU PCS: 41.1 (IQR 31.2–47.1)  No ICU PCS: 46.7 (IQR 38.3–51.9) |
| de Sousa KCA^20^ | *Physiother Res Int*. | Matched case control study | SF-36 | 80 | >12 weeks | Physical functioning PCS: 90 (IQR 66–100)  Physical functioning no PCS: 100 (IQR 86–100)  Physical role limitations PCS: 87.5 (IQR 50–100)  Physical role limitations no PCS: 100 (IQR 100–100)  Bodily pain PCS: 52 (IQR 41–82)  Bodily pain no PCS: 84 (IQR 74–84)  General health perceptions PCS: 60 (IQR 52–67)  General health perceptions no PCS: 66 (IQR 52–80)  Mental health PCS: 70 (IQR 49–92)  Mental health no PCS: 84 (IQR 81–92) |
| Lloyd-Evans PHI^21^ | *BMJ Open Respir Res.* | Pre-post study (intervention after acute COVID) | EQ-5D-5L  EQ-VAS | 110 | median 351 days  (82-457 days) | EQ-VAS pre: 48.8 (SD 19.5)  EQ-VAS post: 59.9 (SD 22.1) |
| Kimmig LM^22^ | *Respir Res.* | Observational cohort study | EQ-VAS | 71 | up to 1 year | EQ-VAS 72.8 (SD 17.7) |
| Daher A^23^ | *Respir Med.* | Observational prospective study | EQ-VAS | 33 | 6 weeks after discharge | EQ-VAS: 63 (IQR 53-80) |
| Carfì A^24^ | *JAMA* | Observational cohort study | EQ-VAS | 143 | 2 months | EQ-VAS 10-point reduction from before acute infection: 44% |
| Garrigues E^25^ | *J Infect*. | Cohort (phone questionnaire) | EQ-5D-5L  EQ-VAS | 120 | 100 days | EQ-VAS: 70.3% (SD 21.5)  EQ-5D-5L index: 0.86 (SD 0.20) |
| Moreno-Perez O^26^ | *J Infect*. | Prospective cohort study | EQ-VAS | 277 | 8-12 weeks | EQ-VAS pre-infection: 90 (IQR 80-100)  EQ-VAS post-infection: 83 (IQR 70-90) |
| Weerahandi H^27^ | *J Gen Intern Med.* | Prospective single health system observational cohort study | PROMIS® Global Health-10 | 161 | 1 month | Physical health pre: 54.3 (SD 9.3)  Mental health pre: 54.3 (SD 7.8)  Physical health post: 43.8 (SD 9.3)  Mental health post: 47.3 (SD 9.3) |
| Arnold DT^28^ | *Thorax* | Prospective cohort study | SF-36 | 110 | 8-12 week | Physical composite score mild 41 (SD 12)  Physical composite score moderate 41 (SD 12)  Physical composite score severe 36 (SD 7)  Mental composite score mild 45 (SD 11)  Mental composite score moderate 46 (SD 11)  Mental composite score severe 40 (SD 17) |
| Logue JK^29^ | *JAMA Net Open* | Longitudinal prospective cohort | EQ-VAS | 177 | median 169 days | EQ-VAS 10-point reduction from before acute infection: 30% |

PCS: Post COVID-19 syndrome, SD: standard deviation

## References

1. Pinzon RT, Wijaya VO, Jody AA, Nunsio PN, Buana RB. Persistent neurological manifestations in long COVID-19 syndrome: A systematic review and meta-analysis*. J Infect Public Health.* 2022;**15**(8):856-869.
2. Lopez-Leon S, Wegman-Ostrosky T, Ayuzo Del Valle NC, et al. Long-COVID in children and adolescents: a systematic review and meta-analyses. *Sci Rep.* 2022 Jun 23;**12**(1):9950.
3. Nguyen NN, Hoang VT, Dao TL, Dudouet P, Eldin C, Gautret P. Clinical patterns of somatic symptoms in patients suffering from post-acute long COVID: a systematic review. *Eur J Clin Microbiol Infect Dis*. 2022;**41**(4):515-545. doi:10.1007/s10096-022-04417-4
4. Alkodaymi MS, Omrani OA, Fawzy NA, et al. Prevalence of post-acute COVID-19 syndrome symptoms at different follow-up periods: a systematic review and meta-analysis. *Clin Microbiol Infect*. 2022;**28**(5):657-666.
5. Ceban F, Ling S, Lui LMW, et al. Fatigue and cognitive impairment in Post-COVID-19 Syndrome: A systematic review and meta-analysis. *Brain Behav Immun*. 2022;**101**:93-135.
6. Renaud-Charest O, Lui LMW, Eskander S, et al. Onset and frequency of depression in post-COVID-19 syndrome: A systematic review. *J Psychiatr Res*. 2021;**144**:129-137.
7. Michelen M, Manoharan L, Elkheir N, et al. Characterising long COVID: a living systematic review. *BMJ Glob Health*. 2021;**6**(9):e005427.
8. Cares-Marambio K, Montenegro-Jiménez Y, Torres-Castro R, et al. Prevalence of potential respiratory symptoms in survivors of hospital admission after coronavirus disease 2019 (COVID-19): A systematic review and meta-analysis. *Chron Respir Dis*. 2021;**18**:14799731211002240.
9. Tsuzuki S, Miyazato Y, Terada M, Morioka S, Ohmagari N, Beutels P. Impact of long-COVID on health-related quality of life in Japanese COVID-19 patients. *Health Qual Life Outcomes.* 2022 Aug 19;**20**(1):125.
10. Malik P, Patel K, Pinto C, et al. Post-acute COVID-19 syndrome (PCS) and health-related quality of life (HRQoL)—A systematic review and meta-analysis. *J Med Virol*. 2022 Jan 1;**94**(1):253–62.
11. Taboada M, Moreno E, Cariñena A, et al. Quality of life, functional status, and persistent symptoms after intensive care of COVID-19 patients. *Br J Anaesth*. 2021 Mar 1;**126**(3):e110–3.
12. Huang C, Huang L, Wang Y, et al. 6-month consequences of COVID-19 in patients discharged from hospital: a cohort study. *Lancet*. 2021 Jan 16;**397**(10270):220–32.
13. Catalán IP, Martí CR, Sota DP, et al. Corticosteroids for COVID-19 symptoms and quality of life at 1 year from admission. *J Med Virol*. 2022 Jan 1;**94**(1):205–10.
14. Tabacof L, Tosto-Mancuso J, Wood J, et al. Post-acute COVID-19 Syndrome Negatively Impacts Physical Function, Cognitive Function, Health-Related Quality of Life, and Participation. Am J Phys *Med Rehabil.* 2022;**101**(1).
15. Poudel AN, Zhu S, Cooper N, et al. Impact of Covid-19 on health-related quality of life of patients: A structured review. *PLoS One*. 2021 Oct 28;**16**(10):e0259164.
16. McFann K, Baxter BA, LaVergne SM, et al. Quality of Life (QoL) Is Reduced in Those with Severe COVID-19 Disease, Post-Acute Sequelae of COVID-19, and Hospitalization in United States Adults from Northern Colorado. *Int J Environ Res Public Health.* 2021 Oct 21;**18**(21):11048.
17. Schouborg LB, Molsted S, Lendorf ME, et al. Risk factors for fatigue and impaired function eight months after hospital admission with COVID-19. *Dan Med J*. 2022 Mar 16;**69**(4):A08210633.
18. Kim Y, Kim S-W, Chang H-H, Kwon KT, Hwang S, Bae S. One Year Follow up of COVID-19 Related Symptoms and Patient Quality of Life: A Prospective Cohort Study. *Yonsei Med J*. 2022 Jun;**63**(6):499–510.
19. Muñoz-Corona C, Gutiérrez-Canales LG, Ortiz-Ledesma C, et al. Quality of life and persistence of COVID-19 symptoms 90 days after hospital discharge. *J Int Med Res*. 2022 Jul 1;**50**(7):03000605221110492.
20. de Sousa KCA, Gardel DG, Lopes AJ. Postural balance and its association with functionality and quality of life in non-hospitalized patients with post-acute COVID-19 syndrome. *Physiother Res Int*. 2022 Oct 1;**27**(4):e1967
21. Lloyd-Evans PHI, Baldwin MM, Daynes E, et al.; Your COVID Recovery® Development Group. Early experiences of the Your COVID Recovery^®^ digital programme for individuals with long COVID. *BMJ Open Respir Res.* 2022 Sep;**9**(1):e001237.
22. Kimmig LM, Rako ZA, Ziegler S, et al. Long-term comprehensive cardiopulmonary phenotyping of COVID-19. *Respir Res.* 2022;**23**(1):263.
23. Daher A, Balfanz P, Cornelissen C, et al. Follow up of patients with severe coronavirus disease 2019 (COVID-19): Pulmonary and extrapulmonary disease sequelae. *Respir Med.* 2020 Nov-Dec;**174**:106197.
24. Carfì A, Bernabei R, Landi F, Group for the GAC-19 P-ACS. Persistent Symptoms in Patients After Acute COVID-19. *JAMA*. 2020 Aug 11;**324**(6):603–5.
25. Garrigues E, Janvier P, Kherabi Y, et al. Post-discharge persistent symptoms and health-related quality of life after hospitalization for COVID-19. *J Infect*. 2020 Dec 1;**81**(6):e4–6.
26. Moreno-Pérez O, Merino E, Leon-Ramirez J-M, et al. Post-acute COVID-19 syndrome. Incidence and risk factors: A Mediterranean cohort study. *J Infect.* 2021 Mar 1;**82**(3):378–83.
27. Weerahandi H, Hochman KA, Simon E, et al. Post-Discharge Health Status and Symptoms in Patients with Severe COVID-19. *J Gen Intern Med.* 2021;**36**(3):738–45.
28. Arnold DT, Hamilton FW, Milne A, et al. Patient outcomes after hospitalisation with COVID-19 and implications for follow up: results from a prospective UK cohort. *Thorax*. 2021 Apr 1;**76**(4):399 LP – 401.
29. Logue JK, Franko NM, McCulloch DJ, et al. Sequelae in Adults at 6 Months After COVID-19 Infection. *JAMA Netw Open*. 2021 Feb 19;**4**(2):e210830–e210830.
