# Supplementary material for: Clinical phenotypes and quality of life to define post-COVID-19 syndrome: a cluster analysis of the multinational, prospective ORCHESTRA cohort
Source: eClinicalMedicine. 2023 Jul 21;62:102107. doi: 10.1016/j.eclinm.2023.102107 (PMC10466236; doi:10.1016/j.eclinm.2023.102107)
Supplement: The ORCHESTRA Study Group [file mmc2.docx]

**The ORCHESTRA study group**

Mariana Nunes Pinho Guedes, Gaia Maccarrone, Maria Diletta Pezzani, Marcella Sibani, Ruth Joanna Davies, Stefania Vitali, Giorgia Franchina, Giorgia Tomassini, Concetta Sciammarella, Riccardo Cecchetto, Davide Gibellini, Chiara Konishi De Toffoli, Giulia Rosini, Chiara Perlini, Marco Meroi, Filippo Cioli Puviani, Daniele Fasan, Claudio Micheletto, Stefania Montemezzi, Nicolò Cardobi, Gianluca Vantini, Gloria Mazzali, Giovanni Stabile, Maddalena Marcanti, Marco Pattaro Zonta, Deborah Calì, Anna Mason, Cinzia Perlini, Paolo Gisondi, Maria Mongardi, Simona Sorbello (University Hospital of Verona); Karin I. Wold, María F. Vincenti-González, Alida C. M. Veloo, Valerie P. R. Harmsma, Daniele Pantano, Margriet van der Meer, Lilli Gard, Erley F. Lizarazo, Marjolein Knoester, Alex W. Friedrich, Hubert G. M. Niesters (University of Groningen); Pierluigi Viale, Domenico Marzolla, Federica Cosentino, Michela Di Chiara, Giacomo Fornaro, Cecilia Bonazzetti, Beatrice Tazza, Alice Toschi, Oana Vetamanu, Maria Eugenia Giacomini, Fabio Trapani, Lorenzo Marconi, Luciano Attard, Sara Tedeschi, Liliana Gabrielli, Tiziana Lazzarotto (University of Bologna); Paula Olivares,^,^ Javier Castilla, Javier Vélez, Virginia Almadana, Lucía Martín-Barrera, Ana Belén Martín-Gutiérrez, David Gutiérrez-Campos, Marta Fernández-Regaña, Ana Silva-Campos, Patricia Fernández-Riejos, M. Isabel García-Sánchez (Hospital Universitario Virgen Macarena); Carla V. Giuliano, Carlota López, Gabriela Neumann, Julieta Camporro, Lautaro de Vedia, Hugo Agugliaro (University of Buenos Aires); Gabriella Scipione, Chiara Dellacasa, Balasubramanian Chandramouli, Silvia Gioiosa, Juan Mata Naranjo, Maurizio Ortali (CINECA Interuniversity Consortium, Bologna); Angelina Konnova, Akshita Gupta, Mathias Smet, An Hotterbeekx, Matilda Berkell (University of Antwerp); Elisa Sicuri (Barcelona Institute for Global Health - ISGlobal); Delphine Bachelet, Lila Bouadma, Minerva Cervantes-Gonzalez, Anissa Chair, Charlotte Charpentier, Léo Chenard, Diane Descamps, Hang Doan, Xavier Duval, Marina Esposito-Farese, Isabelle Hoffmann, Ouifiya Kafif, Quentin Le Hingrat, Sophie Letrou, France Mentré, Marion Schneider, Coralie Tardivon, Jean-Francois Timsit, Sarah Tubiana (Hôpital Bichat, Paris); Amal Abrous, Sandrine Couffin-Cadiergues, Fernanda Dias Da Silva, Hélène Esperou, Ikram Houas, Salma Jaafoura, Aurélie Papadopoulos (Inserm, Paris); Severine Ansart (CHU Brest); Adrien Auvet (CH Dax); Firouzé Bani-Sadr (CHU Reims); L. Bernard (CHU Tours); François Bissuel (CH Thonon les Bains); Elisabeth Botelho-Nevers (CHU Saint Etienne); Damien Bouhour (CH Bourg en Bresse); André Cabié (CHU Martinique); Pauline Caraux Paz (CH Villeneuve St Georges); Christian Chidiac (CHU Lyon); Catherine Chirouze (CHU Besancon); Tomasz Chroboczek (CH Alpes Leman, Contamine sur Arve); Hugues Cordel (Hôpital Avicenne, Bobigny); Roxane Courtois (CH Cholet); Nathalie De Castro (Hôpital Saint Louis, Paris); Sylvain Diamamntis (CH Melun); Jean-Luc Diehl (HEGP, Paris); Felix Djossou (CH Cayenne); Céline Dorival (Inserm, Paris); Olivier Epaulard (CHU Grenoble); Valerie Gaborieau (CH Pau); François Goehringer (CHU Nancy); Marie Gousseff (CH Bretagne Atlantique, Vannes); Simon Jamard (Hôpital Bretonneau, Tours); Cedric Joseph (CHU Amiens); Karine Lacombe (Hôpital Saint Antoine, Paris); Soizic Le Mestre (ANRS-MIE, Paris); Vincent Le Moing (CHU Montpellier); Jean-Daniel Lelievre (Hôpital Mondor, Créteil); Olivier Lesens (CHU Clermont-Ferand); M. Machado (GHEF Marne La Vallée); Mylène Maillet (CH Annecy Genevois); Victoria Manda (Hôpital Lariboisière, Paris); Guillaume Martin-Blondel (CHU Toulouse); Martin Martinot (CH Colmar); Vanina Meysonnier (GH Diaconesses Croix Saint-Simon, Paris); Jean-Michel Molina (Hôpital Saint Louis, Paris); Eric Oziol (CH Beziers); Vincent Pestre (CH Avignon); Lionel Piroth (CHU Dijon); Julien Poissy (CHU Lille); Christian Rabaud (CHU Nancy); François Raffi (CHU Nantes); Blandine Rammaert (CHU Poitiers); Christophe Rapp (Hopital Américain, Neuilly sur Seine); Stanislas Rebaudet (Hopital Europeen, Marseille); Pierre-Marie Roger (CHU Guadeloupe); Damien Roux (Hôpital Louis Mourrier, Colombes); Eric Senneville (CH Tourcoing); Pierre Tattevin (CHU Rennes); Aurélie Wiedemann (Vaccine Research Instiute – VRI, Inserm, Créteil); David Zucman (Hôpital Foch, Suresnes)
